# Milk Lipids as Bioactive Modulators of the Bacterial Proteome: Mechanisms Linking Dairy Management to Microbial Performance

**DOI:** 10.3390/ani16030477

**Published:** 2026-02-03

**Authors:** Anna Maria Ogrodowczyk, Karolina Kowalska, Dominik Kulasik

**Affiliations:** 1 Immunology and Food Microbiology Group, InLife Institute of Animal Reproduction and Food Research of Polish Academy of Sciences, Trylińskiego Str. 18, 10-683 Olsztyn, Poland; 2 Faculty of Biology and Biotechnology, University of Warmia and Mazury, Oczapowskiego Str. 1A, 10-719 Olsztyn, Poland; dkulasik.contact@gmail.com; 3 Doctoral School of Natural Sciences, Adam Mickiewicz University, Uniwersytetu Poznańskiego 6, 61-614 Poznań, Poland

**Keywords:** milk, lipid profile, bacteria, microbial proteins, starter cultures, milk microbiota, animal diet, welfare, intrinsic factors, extrinsic factors

## Abstract

Milk lipids are critical determinants of dairy product quality, human health, and animal welfare. Their composition is shaped by a complex interplay of genetic, dietary, physiological, and environmental factors. These lipids play a pivotal role in modulating the expression and function of bacterial proteins in both indigenous microbiota and starter cultures. However, the mechanistic pathways linking farm-level factors to the microbial phenotypes observed during dairy fermentation remain underexplored. This review synthesizes existing knowledge on how intrinsic and extrinsic factors modify milk production and lipid profiles in dairy animals and indirectly reshape bacterial protein expression. By using a cross-species approach comparing milk lipid metabolism in dairy animals and humans, we bridge critical knowledge gaps to inform future optimization strategies. We examine evidence linking lipid profiles to microbial protein synthesis and localization, with a special focus on the adaptive role of moonlighting proteins. Finally, this review proposes a novel integrative framework linking livestock management practices directly to microbial performance in dairy fermentation. This perspective identifies milk lipids not merely as nutrients, but as bioactive modulators of the bacterial proteome, offering a new paradigm for enhancing dairy safety and functionality.

## Simple Summary

Milk fat is a complex mix of fatty acids and valuable bioactive compounds that significantly impacts animal and human health, but also milk microbiota, which can influence the quality of dairy products. For years, researchers have been trying to pinpoint exactly what modulates fat composition. Initially, studies focused on how an animal’s diet affected its milk fat. However, we now know that is only part of the story. The fat profile is also shaped by genetics, breed, lactation stage, and even the animal’s gut microbes. This intricate web of diet, genetics, and environment creates a unique fat signature in milk. Furthermore, milk fat interacts with the bacteria used to make cheese and yogurt, which is crucial for the final product’s flavor, texture, and safety. However, detailed research on the interactions between milk fat and the resulting bacterial protein profiles is sparse. This review aims to synthesize existing knowledge and fill these gaps. By combining the latest findings in genomics, lipidomics, and epigenetics, we can better understand how to manipulate milk fat profiles. Our goal is to provide insights that will help optimize breeding and feeding strategies, ultimately leading to higher-quality milk and more functional, safer dairy products.

## 1. Introduction

**Figure 1:**
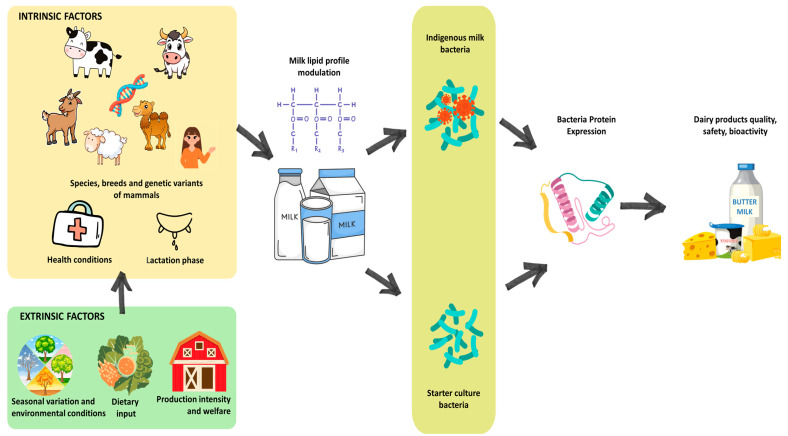
Factors Modulating Milk Lipid Profiles and Subsequent Influence on Bacterial Protein Expression in Dairy Products.

Research on the role of milk lipid profiles (MLPs) has emerged as a critical area of inquiry due to its implications for animal welfare, dairy product quality and human health. Milk lipids, comprising diverse fatty acids (FAs) and liposoluble bioactive compounds like vitamins, influence the nutritional value and technological properties of dairy products [1,2]. Over recent decades, studies have evolved from focusing on dietary and photoperiod effects on milk FA composition [3,4] to integrating genetic, environmental, and physiological factors such as variant, breed, lactation stage, epigenetic changes and animal health, including gastrointestinal tract microbiota composition [5,6,7]. In contrast, the relationship between MLP and the protein expression of both the indigenous milk microbiota (IMM) and the starter cultures used for dairy fermentation represents a complex interplay of biochemical and environmental factors. The conceptual framework underpinning this review, illustrating the pathway from environmental factors to bacterial response via milk lipids, is summarized in Figure 1. Milk constitutes a chemically and structurally dynamic microbial ecosystem enriched in lipids, and its components, including triacylglycerol (TAG), free fatty acids (FFA), phospholipids, sphingolipids, ceramides, and cholesterol, represent a unique modulatory environment that shapes the behavior of resident and inoculated bacteria. Traditionally, lipids have been studied as nutritional substrates, but evidence across proteomics, lipidomics, metabolomics, and transcriptomics indicates that these molecules act as potent regulators of bacterial protein expression, membrane architecture, stress physiology, and metabolic fluxes [8].

Raw milk contains an IMM, including species of lactic acid bacteria (LAB) like *Lactococcus*, *Lactobacillus* (now reclassified into >20 genera), *Bifidobacterium*, *Streptococcus*, and numerous other environmental taxa of accompanying microbiota *Enterococcus*, *Bacillus*, *Clostridium*, *Propionibacterium*, (sometimes potentially pathogenic), which affects the quality of final products [9,10]. These bacteria have evolved unique lipid-responsive phenotypes due to long-term exposure to the fluctuating lipid composition of milk. In contrast, industrial starter cultures have undergone domestication, a process that improves acidification performance and technological stability but narrows ecological adaptability and lipid responsiveness [11].

Understanding how intrinsic factors (genetic, metabolic, and hormonal *i.a.*) and extrinsic factors (diet, intensity of production, and seasonal variation *i.a.*) directly influence lipid profiles and indirectly affect bacterial protein expression is essential for optimizing dairy production and fermentation processes, as well as for ensuring the safety and high bioactivity of dairy products [12,13]. However, the available research literature provides limited direct evidence for these specific mechanistic pathways. This review synthesizes dispersed findings to provide a coherent conceptual framework, addressing a gap in how these relationships are currently organized and interpreted.

Few recent studies have begun to uncover how the modulation of milk’s lipid profile can indirectly influence bacterial protein expression, offering new insights into dairy biotechnology and functional food development [14]. The central mechanism underpinning this phenomenon lies in the dynamic structure of milk fat globules (MFGs) which consist of a triglyceride-rich core enveloped by a membrane composed of phospholipids, cholesterol, and proteins. Intrinsic factors such as milk pH, breed-specific composition, and lactation stage can alter the size, stability, and biochemical makeup of these MFGs [15]. Also, technological processes like homogenization and thermal processing, further modulate the lipid profile.

These changes generate a distinct lipid environment that shapes bacterial responses during fermentation. Through effects on nutrient availability, membrane dynamics, stress signaling, and metabolic regulation, milk lipids modulate bacterial protein expression [14,16]. As a result, the milk lipid profile acts as a biochemical interface linking environmental and compositional factors to microbial gene regulation. Clarifying this pathway provides a foundation for optimizing fermentation processes, improving dairy quality, and developing functional foods with targeted microbial activity. This is particularly relevant given the increasing global demand for functional dairy products enriched with microbial metabolites but also beneficial lipids, particularly polyunsaturated fatty acids (PUFAs) and conjugated linoleic acid (CLA), which underscores the practical and economic significance of this research domain [17,18]. Epidemiological evidence linking enhanced milk FAs profiles to potential reductions in cardiovascular disease risk and other health benefits has further amplified the societal relevance of milk lipid manipulation research [19,20]. Consequently, dairy production systems worldwide have increasingly adopted strategies to optimize MLP, with pasture-based diets and strategic lipid supplementation showing promise in enhancing milk quality while maintaining production efficiency [1,18,21].

While numerous studies demonstrate that dietary interventions can substantially modify milk FA composition, the magnitude and direction of these changes vary depending on feed type, processing method, and animal physiology. Significant knowledge gaps remain regarding the combined influence of intrinsic factors (genetics, breed, lactation phase, health status) and extrinsic factors (mammalian dietary modifications, environmental conditions, animal welfare) modulating milk lipid composition across different milk sources and breeds [2,22,23,24]. While some studies highlight breed-specific lipidomic variations [23,25], others emphasize the dominant role of diet and lactation stage [1,22]. Uncertainties remain regarding the relative contributions of genetics versus nutrition and the extent to which milk lipids can be manipulated without compromising animal health or product functionality [4,26]. These knowledge gaps limit our ability to tailor milk lipid profiles for specific technological or nutritional applications and to fully understand how such profiles influence bacterial protein expression during dairy processing [26,27]. Although it is not known to what extent it is still possible to modify things at the physiological level It is known, that milk lipid composition is shaped by de novo synthesis in the mammary gland and preformed FAs derived from diet and body reserves, with interactions modulated among other things, by lactation phase and breed genetics [5,28].

The conceptual framework of this review integrates the multifactorial determinants of MLP, including genetic selection, physiological status, dietary inputs, and environmental influences [2,24]. That supports the investigation of how these factors collectively influence MLP and their functional roles in modulating bacterial protein expression in dairy applications [26,27]. This review employs a systematic approach to comprehensively evaluate the intrinsic and extrinsic factors that affect MLP, using a comparative analysis across various milk sources and breeds. It also uses narrative reviews of issues indirectly related to the impact of lipids on the microbiota proteome. The review aims to bridge existing knowledge gaps by synthesizing recent advances in lipidomics, genomics, and nutritional interventions, thereby informing breeding and feeding strategies to optimize milk quality and safety, as well as dairy product functionality [1,23,29]. To achieve this, we analyze peer-reviewed studies focusing on dietary, genetic, environmental, and physiological influences on milk lipid composition, leveraging recent advances in lipidomics and proteomics to elucidate the complex interactions and bridge the existing gaps [30,31].

## 2. Materials and Methods

This study is an integrative literature review guided by the question:

“How intrinsic and extrinsic factors indirectly influence protein expression in IMM and starter culture bacteria through direct milk’s lipid profile modulation?”. Relevant studies were retrieved from Scopus, Web of Science, AGRIS, Google Scholar using the keywords: “milk”, “fat composition”, “intrinsic factors”, “extrinsic factors”, and “bacteria proteins”, with the search strategy: milk lipids composition OR milk fat composition OR fatty acids composition AND (animal diet OR animal welfare OR animal gastrointestinal microbiota OR animal production intensity OR animal species OR animal breeds OR animals genetic variants OR animal lactation phase OR animal seasonal variation OR environmental conditions OR animal epigenetic changes OR extrinsic factors OR intrinsic factors) AND indigenous milk bacteria protein expression OR starter cultures bacteria protein expression. Inclusion criteria were original articles published in English between 2000 and 2025, describing the role of milk fat impact on bacterial protein expression by directly affecting their cell membranes, metabolism and signaling. Exclusion criteria comprised studies that did not specify factors’ impact on milk fat composition, did not concern milk obtained for the production of dairy products or did not focus on milk fat macronutrient in the offspring nutrition, were focused on the direct impact of milk macronutrients on human health. The experimental studies were assessed using the CADIMA tool (vers. 2.2.4.2) (https://www.cadima.info/index.php, accessed on 23 August 2025), which evaluates selection, performance, detection, attrition, and reporting biases. Since no quantitative synthesis was possible, the synthesis of the results was done in a narrative form. Study selection involved title, keywords, and abstract screening, followed by full-text review. Studies investigating the impact of lipids on milk microbiota were included in the discussion when relevant.

## 3. Results

### 3.1. Direct Influence of Intrinsic and Extrinsic Factors on Milk Lipid Profiles

This section summarizes current research on the exploration of intrinsic and extrinsic factors, such as dietary modifications, genetic selection, comparative analysis across different milk sources and breeds, environmental conditions, animal welfare, health, and the lactation phase in manipulating milk’s lipid profile. The objective is to examine how milk lipids can modulate bacterial protein expression in dairy applications, integrating studies that examine the multifactorial influences on milk lipid composition. The studies include various ruminant species, breeds, and geographic regions, employing diverse analytical techniques including lipidomics, proteomics, genomics, and metabolomics. This comparative synthesis highlights the integration of genetic, nutritional, environmental, and physiological factors shaping MLP and explores recent evidence on milk lipids’ functional roles in modulating microbial protein expression, thereby contributing to the central research objectives of this review.

**Figure 2:**
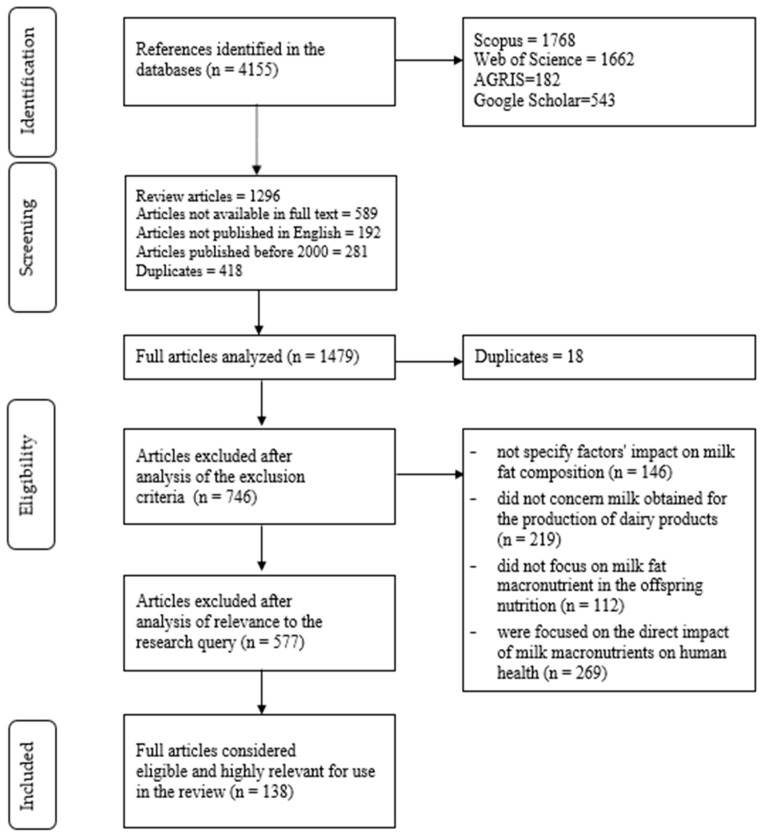
Diagram of study Identification and Screening Workflow.

A total of 4155 articles were obtained from the search in the different databases. Because the search was conducted across multiple databases using broad, interdisciplinary keyword combinations, the initial dataset naturally contained numerous duplicates and non-relevant records. This outcome is further amplified by differences in indexing practices across databases, which often classify lipidomics, microbiology, and dairy science literature under overlapping or inconsistently defined categories. In the initial screening phase, 2776 records were excluded what is detailed in Figure 2 (Screening section), including eliminated article type, language, publication date, and duplication. “After identifying 4155 records, 2776 were excluded during the initial screening phase based on the criteria detailed in Figure 2, including article type, language, publication date, and duplication. The ‘Screening’ bracket in the figure summarizes these eliminations. The full-text assessment of the remaining 1479 publications was then conducted to identify studies that described the relationships between modulators of the milk fat profile and provided a reliable, advanced characterization of milk lipid composition, while also addressing milk fat in the context of human health, animal health, or dairy product manufacturing, rather than focusing on the direct impact of total fat on human health or on chemical applications such as isolation, purification, or analytical method optimization. At this stage, additional records were still excluded due to duplication, but most were removed because they did not assess the milk lipid profile directly; instead, they referred to milk fat only in general terms, reported basic fat content measurements without lipid profiling, and lacked any meaningful connection to microbiota-related outcomes. All of these details are presented in the Eligibility section of Figure 2, where the specific exclusion categories and the corresponding numbers of eliminated papers are provided. After the selection and removal of duplicates and articles that did not meet the inclusion criteria, 138 manuscripts were considered eligible and highly relevant for inclusion in the synthesis. Following the defined selection criteria, the workflow for article inclusion and exclusion, resulting in the final dataset for synthesis, is presented as a PRISMA-compliant flow diagram in Figure 2. Table 1, Tables S1 and S2 show the articles included in the sections dedicated to the impact of intrinsic and extrinsic factors on MLP.

Results of this review are presented according to the author, year, factors and analytical method. Manuscripts that used modern, high-throughput analytical techniques, advanced screening and statistical techniques, and meta-analyses were selected for publication. Table S2 additionally summarizes the selected manuscripts describing rumen bio-hydrogenation modulation impact, the impact of tested factors on milk FA composition and on the health and technological value of milk.

#### 3.1.1. Dietary Influence

A substantial body of research demonstrates the pivotal role of dietary interventions in modulating MLP [1,2,4,14,19,21,22,26,27,28,29,30,31,32,33,34,35,36,37,38,39,40,41,42,43,44,45,46,47,48,49,50,51,52,53,54,55,56,57,58,59,60,115,116,139,140,141,142,144,145]. A collective body of over 75 studies has demonstrated that both the type of feed and the nature of dietary supplements significantly influence the composition and functionality of milk lipids. Dietary modifications, such as pasture-based feeding, oilseed supplementation, and varying forage types, have been demonstrated to significantly alter the FA composition of milk [32]. Specifically, these strategies tend to increase the proportion of beneficial PUFA while reducing saturated fatty acids (SFAs), contributing to improved nutritional quality of milk [1,33,43,117]. Meta-analyses further confirm that SFA-rich diets enhance milk fat production, whereas diets rich in UFA often suppress it, emphasizing the importance of FA profiles in shaping milk lipid output [18,34]. Many studies reported increased concentrations of beneficial FAs such as CLA, omega-3 PUFA, vaccenic acid, and monounsaturated fatty acids (MUFAs) following dietary intervention [61,91,117]. The application of oilseed and protected fat supplementation consistently achieves the nutritional objective of reducing SFA, with a particularly marked decrease observed in palmitic acid [37,39,42,43,45,69,82,92,122]. However, this modification, is frequently accompanied by a major limitation: milk fat depression (MFD). This undesirable reduction in the overall milk fat content is mechanistically linked to the presence of specific FA isomers or complex shifts in rumen microbial activity resulting from the dietary changes [47,62]. Consequently, optimizing nutritional strategies necessitates a precise optimization of dietary strategies between maximizing beneficial FA enrichment and mitigating the economic and production risks associated with MFD.

Some studies extend these findings to other lipid classes, revealing that dietary interventions also affect triglycerides and phospholipids, which are crucial for milk’s functional properties [1,33]. 52 of the referred studies have explored a wide array of dietary supplements—including oilseeds, plant oils, marine lipids, agro-industrial by-products, and protected fats—as tools to manipulate milk FA profiles [82,91,117]. The effectiveness of these supplements is closely tied to their processing methods (e.g., extrusion, encapsulation) and inclusion levels, which can significantly influence the extent of lipid modulation [32,91,119]. Innovative approaches have also examined unconventional feed sources such as tanniferous plants, algae, and agro-industrial by-products. These alternatives show promise in enhancing MLP without negatively impacting overall milk yield [46,57,146].

#### 3.1.2. Genetic Variation

Genetic variation represents one of the most influential and stable determinants of milk fat composition, influencing both de novo synthesis and the distribution of individual FA classes. Breed-specific traits and polymorphisms in key lipogenic genes shape distinct milk lipid profiles, with direct consequences for nutritional quality and technological functionality. The regulatory mechanisms described here are presented briefly to provide essential context for understanding how lipid-mediated signals can indirectly influence bacterial protein expression within the milk environment.

##### Interspecies Variation

Among the selected studies, 17 described the dominant role of interspecies variability. The concentration of total fat in milk exhibits the most dramatic interspecies variation, ranging from less than 1.5% in equids (mares, donkeys) to over 30% in many marine mammals (seals, whales), where rapid energy deposition and thermoregulation are critical [1,48,147,148,149,150]. The latter two milk sources are not used in commercial dairy production due to major ethical, practical, and safety issues, despite concepts like “Deep Sea Dairy” exploring its potential as an innovative, sustainable future source if ethical harvesting or synthesis could be managed. Beyond total quantity, the chain length distribution varies significantly [30,48,87,148,150]. Short- and medium-chain fatty acids (SCFA and MCFA): milk from caprine (goats) and ovine (sheep) species is notably richer in C6:0 (caproic), C8:0 (caprylic), and C10:0 (capric) FAs compared to bovine milk. These MCFA are rapidly digested and absorbed, offering distinct metabolic advantages. Long-chain fatty acids (LCFA): bovine milk typically shows high proportions of C16:0 (palmitic acid) and C18:0 (stearic acid), contributing substantially to the SFA fraction [95,151]. All these compositional differences additionally affect the microstructure as well as the rheological and geometrical properties of MFGs in milk from different animal species [118].

##### Breed and Genetic Variation

An increasing number of studies, approximately 20 studies, have highlighted breed-specific differences in milk lipid composition but also identified genetic markers of diversity. Key genetic markers such as *DGAT1*, stearoyl-CoA desaturase (*SCD1*), and several novel candidate genes have been identified as key regulators of FA synthesis and overall lipid profiles in milk [35,66,75]. Also, β-casein *CSN2* genotype A2A2 was concluded to be positively correlated with PUFA, omega-3 and omega-6 and lower SFA content [76,96]. While the effects of breed on milk lipids are generally moderate, they remain statistically significant. Notably, certain native and local breeds exhibit distinct lipid characteristics compared to mainstream commercial breeds, with genetic variation contributing meaningfully to the heritability of milk FA traits [5,44,69,73,85,86,88,89]. Complementary proteomic and transcriptomic analyses have further revealed breed-related differences in milk protein composition and gene expression patterns associated with lipid metabolism, offering deeper insights into the molecular underpinnings of these variations [26,27,67].

#### 3.1.3. Epigenetic Mechanisms

**Figure 3:**
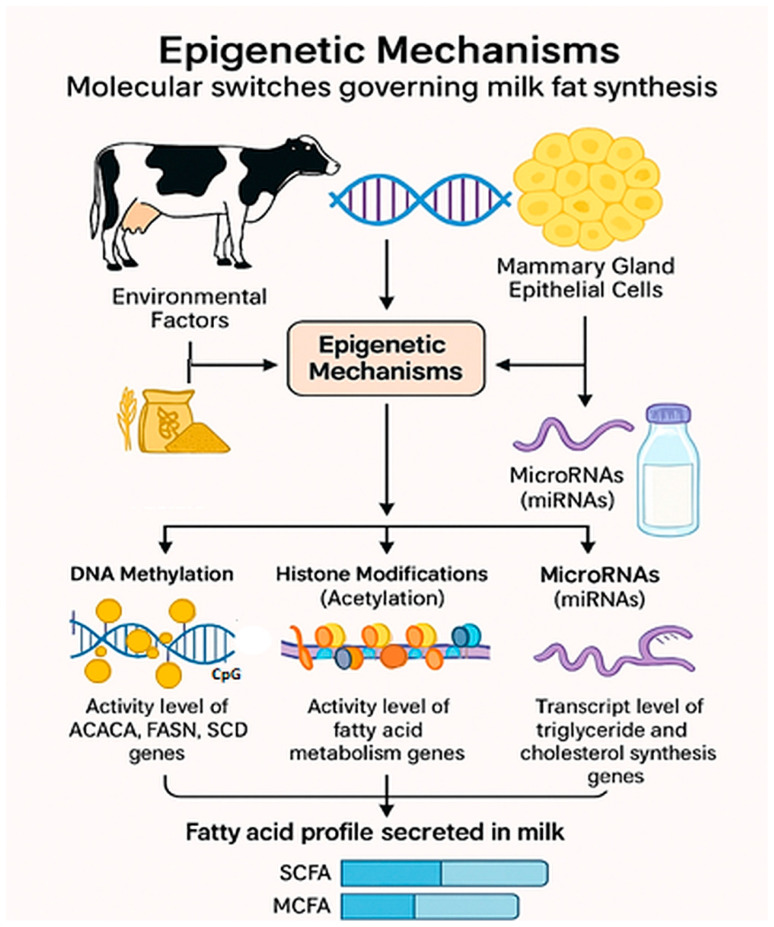
Epigenetic mechanisms of milk fat modulation.

While the genetic makeup of a dairy animal sets the potential for milk fat content, epigenetic modifications, heritable changes in gene function that do not involve alterations to the DNA sequence, provide the mechanistic link between environmental factors (e.g., nutrition and metabolic state) and the actual FA profile secreted into the milk (Figure 3). These mechanisms function as regulatory mechanisms controlling, determining the accessibility and expression levels of the lipogenic mechanisms within the mammary epithelial cells [65].

##### DNA Methylation

Recent research highlights DNA methylation as a key regulator of milk fat production in cows. This process, involving the addition of methyl groups to CpG sites in gene promoter regions, typically silences gene expression. Whole-genome studies on bovine mammary tissue have identified hundreds of differentially methylated CpG sites (DMCs) between cows producing milk with high versus low fat and protein content [65], especially near genes involved in lipid metabolism.

Methylation of lipogenic gene promoters, such as acetyl-CoA carboxylase, fatty acid synthase (FASN), and SCD, directly affects de novo FA synthesis. Elevated methylation, often induced by dietary imbalances (e.g., high-concentrate diets), suppresses these genes, reducing SCFA and MCFA levels and altering milk lipid composition [64,77].

##### Histone Modifications

Histone modifications (acetylation and methylation) are a dynamic layer of epigenetic control that regulates gene expression by altering chromatin accessibility, dictating how tightly DNA is packed. This process is critically dependent on nutrient availability: methyl donors like methionine, choline, and folate dictate the supply of S-adenosylmethionine (SAM), which is vital for modification [78]. For instance, omega-3 fatty acids promote histone acetylation, which increases chromatin accessibility to boost the transcription of lipid metabolism genes, leading to a milk fat profile enriched in beneficial unsaturated fats [79].

##### MicroRNAs (miRNAs)

MicroRNAs (miRNAs) are small, non-coding RNA molecules that provide post-transcriptional epigenetic control by binding to and silencing target messenger RNA (mRNA) sequences. Their expression is directly influenced by dietary FAs, allowing them to regulate key lipid metabolic pathways, including triglyceride and cholesterol synthesis. Some of miRNAs like miR-23a, miR-27b, miR-103, and miR-200a can synergistically regulate milk fat synthesis in mammary gland epithelial cells [81]. Another mechanism acts as a rapid molecular brake on lipogenesis; for example, the dysregulation of miR-200c is specifically associated with diet-induced MFD [80].

#### 3.1.4. Environmental and Physiological Effects

**Figure 4:**
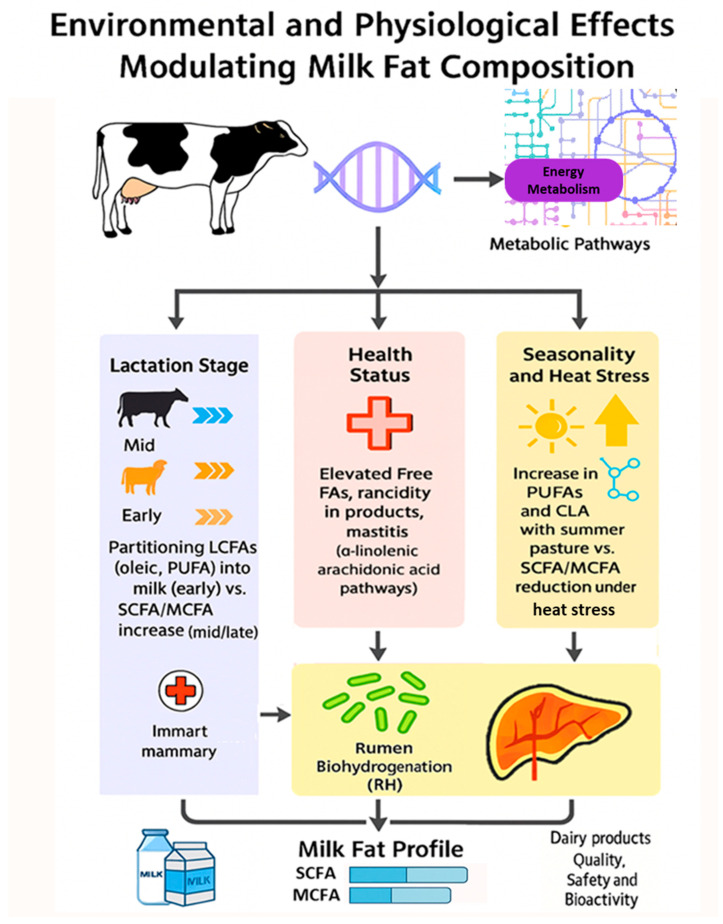
Environmental and physiological effects of milk fat modulation.

Environmental and physiological factors exert a fundamental and often dominant influence on the MLP, modulating it primarily through the intricate regulation of mammals’ metabolism Figure 4. Both physiological and environmental factors ultimately converge on the metabolic pathways responsible for FA synthesis and mobilization, dictating the final nutritional quality of the milk.

##### Endogenous Factor—Lactation Stage

The lactation stage is consistently identified as the strongest intrinsic factor determining the milk FA composition, with early, mid, and late phases showing distinct lipid quality indices [1,22,28]. These profile changes are driven by major metabolic shifts tied directly to the animal’s energy status [30,36,63,72,74]. During the early lactation phase, which is frequently associated with a state of negative energy balance, the cow intensifies the mobilization of its body fat reserves [59]. This metabolic response results in an increased partitioning of LCFA into milk, particularly oleic acid (C 18:1) and PUFA derived from adipose tissue breakdown, serving as a direct reflection of the animal’s health and metabolic status [28,30]. As the cow transitions into mid and late lactation, energy balance stabilizes, and the proportion of SCFA and MCFA, synthesized de novo in the mammary gland gradually increases, indicating the restoration of energetic equilibrium and optimization of de novo milk fat synthesis [1,22].

##### Endogenous Factor—Health Status

The physiological status and health of the lactating mammal introduce influential factors that modulate milk lipid characteristics at the cellular and systemic level. Mastitis, an inflammatory condition of the mammary gland common in dairy species, is a major factor compromising milk quality [59,97,98,145,152]. The inflammation increases the permeability of the mammary epithelium, leading directly to a substantial and consistent rise in total FFA [97,123]. This increase results from the elevated activity of lipolytic enzymes, such as lipoprotein lipase (LPL), within the inflamed tissue, results in accelerated triglyceride hydrolysis.

While the overall FA profile may exhibit subtle changes in saturation or chain length, modern lipidomics confirms that mastitis is correlated with abnormal lipid metabolism, specifically affecting pathways related to α-linolenic and arachidonic acid synthesis [29]. Furthermore, this elevated FFA content is detrimental to the quality of derived dairy products, notably accelerating lipolysis and leading to rancidity in aged cheese [97]. Beyond local inflammation, the gut microbiome balance of the mother holds significant systemic influence. Research established an axis: the mother’s dietary factors influence her systemic metabolism and gut microbiota, which in turn alter the supply of FA precursors transferred to the milk [99,100]. The FA profile of the milk itself (e.g., specific long-chain PUFAs) directly shapes the colonization and function of the offspring’s developing gut microbiome [101].

##### Environmental Modulation—Seasonality and Heat Stress

Furthermore, external seasonal and environmental conditions modulate the MLP, often interacting complexly with diet and breed effects [25,28,89,90,124,139]. The transition to summer grazing, for instance, where fresh pasture is naturally rich in omega-3 fatty acid precursors like α-linolenic acid, leads to a significant elevation in milk PUFA content and CLA, thereby enhancing the milk’s nutritional quality [25]. Conversely, high ambient temperatures can induce heat stress, resulting in decreased dry matter intake (DMI) and subsequent undesirable changes in the MLP, often characterized by a reduction in SCFA and MCFA content as the organism conserves energy [28,124].

##### The Mediating Mechanism—Rumen Biohydrogenation

Regardless of their source, whether mobilized from body fat or absorbed from the gut, these endogenous and exogenous FAs ultimately pass through the RH. Numerous studies highlight the critical role of rumen microbial metabolism in biohydrogenating UFA, a process that severely limits their effective transfer to the milk fat [6,18,38,52,93,115,116,119]. Consequently, effective modulation of milk FA profiles requires targeted interventions to bypass or modulate this microbial process. Strategies such as feeding protected fats, altering forage type, and utilizing plant secondary compounds have been shown to modulate RH pathways, thereby increasing the outflow and subsequent incorporation of beneficial UFA into milk [32,38,93,119]. The inherent complexity and variability of these RH processes across different feeding systems and animals, however, emphasize the need for continued research to optimize these interventions effectively and predictably [31,93,153].

### 3.2. Influence of Milk Lipid Profiles on Bacterial Proteins Expression

The relationship between the complex milk matrix and its resident microbiota is a central theme in dairy science, defining product quality, safety, and functional properties. In this dynamic environment, interactions between milk lipids and bacterial cells shape key proteomic responses that support microbial adaptation and metabolic activity. LAB in fermented products expresses varied protein sets under different environmental conditions. Proteins may be constitutive, with high homology across species, or expressed only under particular situations. Many proteins are moonlighting, performing multiple functions depending on the cell’s physiological state and environmental conditions. Bacterial protein synthesis depends on ancestral DNA sequences, metabolic, and stress-response pathways. Lipids modulate bacterial proteomes through defined mechanisms, including changes in membrane fluidity, lipid-dependent signaling, and the activation of adaptive stress responses. These processes collectively reshape the expression of metabolic, structural, and surface-associated proteins. In milk-bacteria interactions, milk lipids serve not only as an energy source but also as substrates that bacteria can both consume and synthesize, thereby influencing proteomic outcomes [125,126]. Understanding how both intrinsic milk properties and extrinsic processing factors influence bacterial protein expression through this lipid modulation is essential for optimizing industrial dairy fermentation, enhancing probiotic functionality, and controlling the native microbiota.

Lipids reshape bacterial proteomes through three primary mechanisms: (a) substrate competition in FA metabolism, (b) biophysical modulation of membrane environments, and (c) enzymatic lipid modifications that create regulatory feedback loops [102,103,104,105,127]. In LAB, analogously to other bacteria, these mechanisms may drive coordinated changes in stress response proteins, secretion machinery, transport systems, and metabolic enzymes, with direct implications for survival traits including bile tolerance, acid resistance, pH-sensitivity and desiccation survival [128,129,130]. The evidence reveals dose-dependent effects, strain-specific responses, and intricate mechanisms involving membrane incorporation, metabolic regulation, and adaptive responses to environmental stress.

#### 3.2.1. Bacterial Protein Synthesis

##### Constitutive Proteins, Expression and Homology

Diverse bacterial species produce identical proteins either constitutively or inducibly [106,107]. Prokaryotic proteomes contain tens of thousands of proteins, reflecting the wide range of functions governed by a relatively limited number of genes [108,109]. Within this dynamic framework, regulated protein expression represents a fundamental instrument for cellular adaptation [110,131]. Proteins are classified as ‘constitutive’ or ‘induced’ based on their target function and patterns of expression [107,132]. Essential genes encode some proteins and are similar in sequence and structure across numerous organisms; this phenomenon is described as homology [133,134]. Homologous proteins that exist in different species derive, through evolutionary descent, from a common ancestor and describe phylogenetic relationships [135,136]. Furthermore, diverse proteins can have similar functions, creating relations that transcend genetic homology and define ‘functional homology’ or analogies [110,137]. At the gene level, protein sequences can be described as: (a) orthologous if they have diverged after speciation events [138]; (b) paralogous if they arose from gene duplication [138]; and (c) xenologs if transferred between organisms through genetic transfer [111,112]. The category of ‘constitutive protein’ refers to all those continuously produced in the cell at fairly constant levels, independent of the environment, and includes the bulk of cytosolic proteins [106,132].

##### Moonlighting Proteins

Moonlighting proteins, for example, perform multiple physiologically-relevant biochemical or biophysical functions [113,114]. Multifunctional proteins capable of performing distinct, unrelated roles have emerged as a component of bacterial regulatory and metabolic systems [154]. Among the best-characterized examples are constitutive ribosomal proteins, which, in addition to their primary role in translation, participate in gene regulation, stress responses, and interactions with the extracellular environment [155,156,157]. The high sequence homology of these proteins, conserved both within and across bacterial species, indicates strong evolutionary pressure to retain additional, noncanonical functions. This conservation supports the concept that existing protein scaffolds can be adapted to perform additional cellular functions without requiring extensive genomic innovation [157]. This phenomenon becomes particularly relevant in LAB, where modifications in membrane lipid composition and broader metabolic responses to environmental conditions indirectly influence the expression and activity of numerous cellular proteins [158,159,160]. Although the relationships between constitutive proteins and the broader proteome are not yet fully understood, growing evidence suggests that physicochemical changes in the cell membrane can modulate both ribosomal activity and moonlighting behavior—by altering localization, folding states, or ligand interactions of multifunctional proteins [158,160]. Under this model, bacterial adaptation does not rely solely on transcriptional reprogramming or genomic mutations. Instead, bacteria capitalize on the latent functional versatility of existing proteins.

A direct connection between ribosomal proteins and moonlighting proteins has been well documented, particularly in the context of regulatory and antimicrobial activities [161,162,163]. Certain ribosomal proteins exhibit antimicrobial properties or participate in stress-induced gene regulation—functions enabled not by changes in amino acid sequence but by intrinsic conformational flexibility and context-dependent interactions [156,161]. Such off-ribosomal functions provide energetically efficient adaptive strategies: cells need not produce additional dedicated proteins but rather redeploy those already present in the translational apparatus. Moonlighting proteins expand functional proteome capacity without requiring changes at the nucleotide level, making them a powerful means of increasing protein functional diversity [164]. This characteristic underlies one of the most widely accepted explanations for how membrane lipid profiles can indirectly influence the expression or activity of seemingly unrelated proteins in bacterial cultures [158,159,160]. By affecting the cellular environment in which moonlighting proteins operate, lipid conditions can modulate their secondary roles, leading to substantial shifts in the protein profile. In summary, moonlighting proteins serve as a pivotal interface between the stable bacterial genome and the dynamically regulated proteome. By combining constitutive expression with multifunctional potential, they provide bacteria with a rapid, cost-efficient mechanism for environmental adaptation, ultimately enhancing functional flexibility without requiring additional genetic resources.

##### Scale of Microbial Protein Synthesis

Quantitative proteomic studies show that microbial cells are capable of producing vast numbers of protein molecules. Yeast cells express approximately from 2000 to 6000 distinct proteins and contain tens of millions (approximately 4.2 × 10^7^) of total protein molecules copies per cell, highlighting the scale of eukaryotic proteome complexity [165,166]. Bacteria, though smaller, exhibit similarly dense proteomes, with many proteins, such as enolase, displaying strong structural and functional homology to their yeast counterparts [154,167,168,169]. In species such as *Escherichia coli*, total protein mass typically ranges from 100–300 femtograms per cell, depending on growth conditions, providing a benchmark for estimating bacterial biosynthetic capacity [165,169]. Conserved bacterial proteins constitute a major fraction of this mass and form the metabolic backbone of many taxa. In LAB, proteome composition is highly sensitive to both taxonomic identity and environmental context [170,171,172]. Factors such as growth medium, nutrient availability, pH, and redox state have all been shown to substantially shift protein expression patterns, particularly in *Lactobacillus* and *Lactococcus* species (including widely studied *L. rhamnosus* GG) cultivated in various matrices, including whey and cheese-like substrates [11,171,172].

Because these examples illustrate how diverse environmental cues shape LAB proteomes, it is important to highlight another key determinant relevant specifically to dairy systems: the lipid environment of milk. Milk fats and their constituent FAs influence membrane fluidity, cellular energy metabolism, and stress tolerance, which in turn modify translational activity and the scale of protein production. FA composition can alter the balance between anabolic and stress-responsive proteins, reflecting known links between membrane lipid profiles and bacterial proteomic remodeling [11,125,126,170,173]. These lipid-mediated effects are central to dairy fermentation. LAB must maintain efficient carbohydrate metabolism, acidification, and proteolysis while adapting to the physicochemical constraints of MFGs and varying FA concentrations. Proteomic adaptations under these conditions support not only growth but also product safety and fermentation performance, as demonstrated in proteome-level analyses of LAB cultured under dairy-relevant environmental pressures [11,94,173,174]. These findings indicate that LAB maintain a robust yet highly adaptable protein synthesis apparatus. Their proteomes are shaped by both conserved cellular requirements and by environmental cues, particularly those derived from milk lipids, which jointly determine the scale and specificity of protein production during dairy fermentation.

#### 3.2.2. Milk Lipid Composition and Its Mechanistic Roles in Bacterial Modulation

Milk lipids form a chemically rich and structurally diverse group of molecules that profoundly influence the physiology of LAB and the native milk microbiota. Each lipid class contributes distinct mechanistic effects that shape microbial adaptation and ecological behavior.

##### Triacylglycerols

TAG, which constitute roughly 98% of all milk lipids, do not directly affect bacterial cells until they undergo hydrolysis by bovine lipases, native microbial lipases, or LAB-associated esterases. This process releases FFA as well as mono- and diglycerides, which then exert strong effects on bacterial membranes. UFA such as oleic (18:1), linoleic (18:2), and α-linolenic (18:3) acid disrupt lipid packing and alter membrane fluidity due to their cis-double bonds [127,175]. At high concentrations, these compounds induces lipid stress that leads to membrane leakage, depolarization, destabilization of the proton motive force, and activation of ATPase systems and envelope stress regulons [176]. These effects are especially pronounced in strains with low cyclopropane fatty-acid synthase (CFAS) activity or limited ability to cyclopropanate UFA. FFAs also function as metabolic regulators by inhibiting FASII fatty-acid synthesis pathways, serving as substrates for enzymatic modifications such as hydration, and acting as signals for transcriptional regulators [105,130,177]. Native milk microbiota typically show greater tolerance to these molecules and a broader capacity for their utilization than starter LAB [176,178].

##### Free Fatty Acids (FFAs)

FFA represents the most directly bioactive lipid class for bacteria. The effects of lipids and FAs are highly dependent on concentration and bacterial identity [179]. Low FA concentrations (e.g., 5–10 μg/mL) generally promote growth, whereas high concentrations (e.g., 20–40 μg/mL) often prove inhibitory [175]. Depending on chain length and saturation, SCFA (C4–C6) penetrate membranes and acidify the cytoplasm, whereas MCFA and LCFA interfere with membrane physicochemistry and protein function. PUFA can additionally induce oxidative stress because of their susceptibility to peroxidation. When UFA insert into membranes, they increase lateral lipid mobility, reduce packing density, induce curvature stress, lower the membrane phase transition temperature, and increase permeability to ions (H^+^, K^+^, Na^+^). These changes trigger enhanced expression of F_0_F_1_-ATPase complexes [176], increased production of membrane-associated chaperones such as DnaK and GroEL [180], and reorganization of surface proteins involved in adhesion and interaction with the surrounding environment [178]. To counteract FFA toxicity, bacteria employ efflux pumps, including ABC transporters [181], convert UFA into cyclopropane derivatives via CFA synthase, sequester surplus FA within neutral lipid reservoirs, or activate oxidative detoxification pathways such as thioredoxin and methionine sulfoxide reductase systems [103].

##### Phospholipids

Phospholipids of the milk fat globule membrane (MFGM) also play significant roles in modulating bacterial behavior. Phosphatidylcholine is a zwitterionic, bulk-forming lipid; phosphatidylethanolamine promotes negative curvature; phosphatidylserine is negatively charged, signal-active lipid and phosphatidylinositol, despite it is minor in abundance but plays a regulatory function. These lipids influence surface polarity, hydrophobicity, and the exposure of bacterial surface proteins. They help stabilize membranes during acid and cold stress [8,176] and anchor proteins that mediate adhesion and co-aggregation [156,178]. Exposure to MFGM phospholipids has been shown to increase the expression of adhesion-associated proteins, ATPases crucial for pH homeostasis, cryoprotectants, and stress-related chaperones. Phosphatidylethanolamine and phosphatidylserine strongly influence envelope stress regulons, while phosphatidylcholine tends to favor optimization of membrane fluidity [176]. Native strains display more extensive remodeling of their surface proteome in response to these lipids and rely on a wider repertoire of lipid-modifying enzymes, whereas starter LAB respond more predictably and less dynamically [182].

##### Sphingolipids

Although they constitute less than 1% of milk lipids, sphingolipids exert disproportionately strong regulatory effects due to their saturated long-chain structure and their ability to form rigid microdomains resembling lipid rafts with cholesterol [183,184]. These molecules can modulate protein anchoring, alter bacterial surface hydrophobicity, influence adhesion to host epithelial glycans, and shape immune interactions that are key to colonization processes [185].

##### Distinct Lipid-Responsiveness of Native Microbiota vs. Starter Strains

Importantly, native milk microbiota demonstrate a far more extensive capacity to metabolize and adapt to milk lipids than industrial starter cultures. Native strains tend to possess richer repertoires of lipases, phospholipases, and membrane-remodeling enzymes, allowing them to interact with TAG, phospholipids, and sphingolipids through more complex metabolic pathways [178,180,186]. Their proteomic responses under lipid exposure include broader remodeling of membrane proteins, a larger spectrum of induced chaperones and stress enzymes, and a more flexible metabolic rewiring. Starter cultures, by contrast, have undergone metabolic reduction resulting from domestication. Their lipid-responsive behaviors are more predictable but significantly narrower, as selection for acidification efficiency and technological reliability has reduced adaptive plasticity [187]. Consequently, starter cultures show attenuated responses to MFGM lipids, less efficient fatty acid incorporation, and more conservative proteomic shifts [176,186]. To consolidate species- and strain-specific responses, Table 2 summarizes the currently available evidence on how different classes of milk lipids modulate bacterial physiology. These findings highlight clear distinctions between native milk microbiota and industrial starter cultures in their lipid-responsive proteomic and membrane remodeling capacities.

#### 3.2.3. Molecular Mechanisms of Milk Lipids-Bacterial Proteome Interaction

The modulation of the bacterial proteome by milk lipids operates through several interrelated and highly precise molecular mechanisms, transcending simple nutritional uptake.

##### Mechanistic Membrane Remodeling Induced by Milk Lipids

Milk lipids exert profound effects on bacterial cell envelope architecture by integrating directly into lipid bilayers and modulating their physical properties.

**Figure 5:**
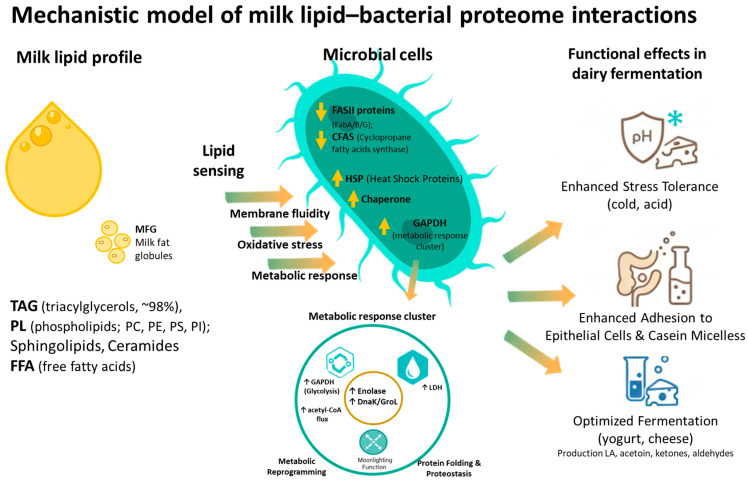
Mechanistic model of lipid–bacteria interactions. ↑ increased effect, ↓ decreased effect, * cold symbole.

Native milk FA, both saturated and unsaturated, embed into the membrane, altering acyl-chain composition and disrupting native packing density. UFA (e.g., oleic, linoleic, ALA) introduce conformational bends, increasing fluidity and inducing curvature strain, whereas saturated fatty acids reinforce ordering and reduce lateral mobility [180,188]. Proteomic data from *Limosilactobacillus* (formerly *Lactobacillus*) *reuteri*, *Lacticaseibacillus casei*, and other LAB indicate that exposure to MFGM lipids and FFA modifies membrane viscosity, local microdomain organization, and protein accessibility [176,183]. One of the most consistent molecular outcomes is suppression of FASII enzymes, including FabA, FabB, FabG, and FabD [177] (Figure 5). This effect emerges from feedback inhibition as exogenous FAs accumulate, decreasing the need for endogenous synthesis as shown by membrane proteomics of lipid-adapted LAB [130].

The curtailment of endogenous FASII reprograms acetyl-CoA trafficking, alters Nicotinamide adenine dinucleotide phosphate (NADPH) utilization, and precipitates downstream modifications in central metabolism and protein expression. The resulting decrease in saturated endogenous FA reduces membrane cohesion, requiring activation of compensatory pathways. In response to these alterations, LAB activate H+-pumping F_0_F_1_-ATPase, which mitigates proton leakage through lipid-disordered bilayers. Proteomic datasets show substantial increases in subunits AtpA, AtpD, and AtpH of ATP complex abundance after lipid exposure, particularly in native strains, which deploy a broader isozyme response [176]. The shift in lipid headgroup distribution, characterized by increased phosphatidylglycerol and reduced phosphatidylethanolamine, may enhance membrane fluidity as a compensatory response to the altered membrane composition what was tested in *Bacillus subtilis* [190]. Chaperone complexes, especially DnaK–DnaJ–GrpE and GroEL–GroES, also increase markedly. These systems are essential for preventing misfolding of membrane-associated proteins destabilized by lipid-dependent changes in bilayer fluidity [180]. Native microbiota exhibit stronger and more diversified chaperone induction than starter cultures, reflecting their broader adaptive elasticity. Simultaneously, the altered lipid microenvironment stimulates the remodeling of transporter systems, particularly ABC transporters, which are critical for fatty-acid uptake, efflux, and detoxification [174]. These systems help regulate intracellular lipid pools and prevent the accumulation of potentially cytotoxic LCFA, thereby maintaining membrane integrity. Lipid-responsive regulatory mechanisms are relevant not only for starter cultures, but also for accompanying microbiota, including opportunistic or sporadically detected pathogenic taxas, that persist in artisanal and spontaneously fermented dairy products. Their ability to remodel proteomic pathways in response to milk lipid composition underscores the need to consider both beneficial and undesirable taxa when evaluating lipid–microbe interactions in dairy environments. Lipid-dependent regulation of bacterial protein expression, as exemplified by phospholipid-driven shifts in two-component systems, protease production and iron-uptake pathways in *Pseudomonas aeruginosa*, illustrates how membrane composition can regulate global adaptive responses in bacteria [191]. Furthermore, surface adhesion proteins, many of which are anchored in or supported by the membrane, undergo notable reorganization under lipid remodeling. Their altered exposure and distribution, often influenced by MFGM phospholipids [176], modify bacterial interactions with milk components, such as casein micelles, and enhance or facilitate adhesion to host epithelial tissues.

##### Lipid-Induced Metabolic Reprogramming

**Figure 6:**
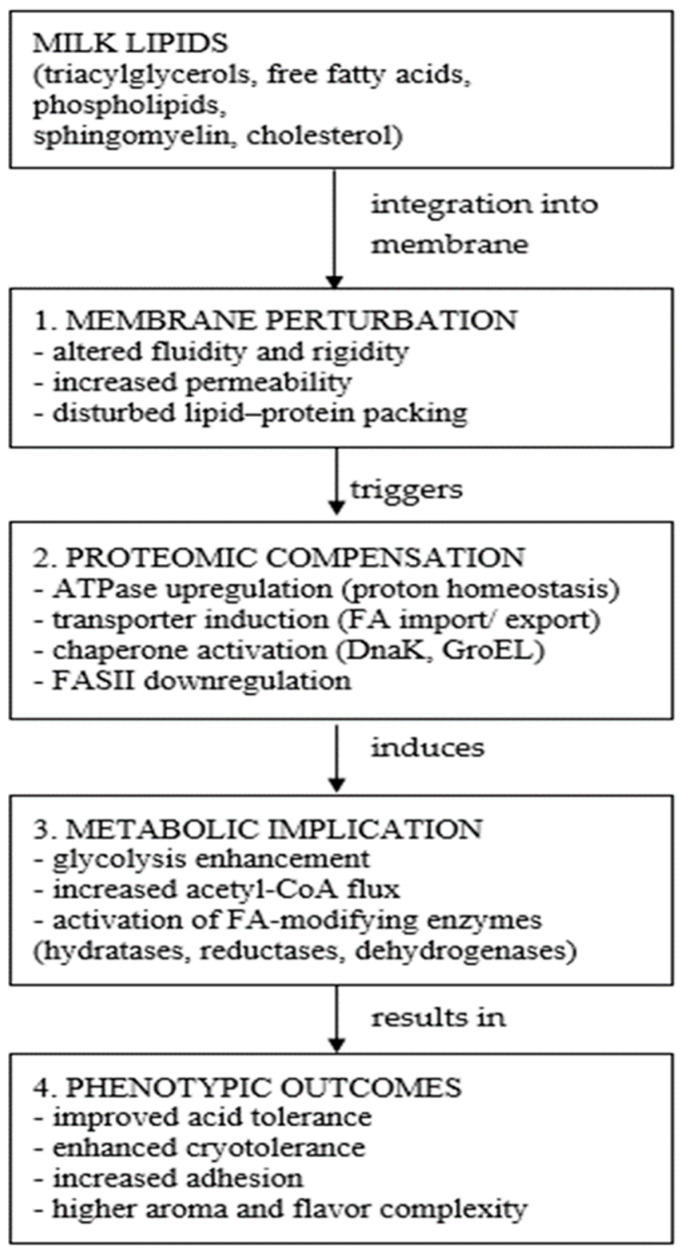
Sequential Cellular Responses to Milk Lipid Integration in LAB.

The integration of exogenous lipids and fatty acids at the membrane level induces a fundamental cascade of metabolic reprogramming within bacterial cells. This central metabolic shift involves the suppression of endogenous FASII pathways, leading to the necessary redirection of the acetyl-CoA carbon flux toward alternative metabolic and stress-response systems [130]. Lipid exposure induces membrane perturbation, which in turn leads to the activation of proton-pumping H^+^-ATPases to maintain intracellular pH homeostasis. This elevated activity increases the bacterial energy demand. To meet this energetic cost, LAB strongly upregulate key glycolytic enzymes, resulting in accelerated glycolysis and higher ATP production [189]. Observed proteomic shifts consistently include the upregulation of glyceraldehyde-3-phosphate dehydrogenase (GAPDH), phosphoglycerate kinase (PGK) and enolase [102,176]. This results in an accelerated conversion of sugars to pyruvate, effectively compensating for the energetic pressure caused by lipid-induced membrane stress [174]. The suppression of endogenous FASII (which normally consumes acetyl-CoA) liberates the acetyl-CoA pool. Under a high exogenous lipid load, this pool is primarily rerouted toward energy production and FA modification pathways. Native strains display a significantly broader metabolic repertoire, often exploiting the lipid environment for survival. They exhibit partial β-oxidation–like activities and induce enzymes specialized in detoxifying of PUFA [103]. A striking example is the induction of linoleate 10-hydratase in strains like *Lactiplantibacillus plantarum*. This enzyme converts linoleic acid into hydroxy-fatty acids, a pathway associated with enhanced gastrointestinal robustness and high lipid exploitation capabilities typically absent in industrial starter strains. To integrate the observed metabolic and proteomic changes, a comprehensive overview of the proposed milk lipids–bacteria interactions and regulatory pathways is provided in Figure 6.

##### Proteostasis and Stress Response Systems Activated by Lipids

Lipid-induced membrane perturbation triggers canonical and specialized stress responses. Heat-shock proteins DnaK and GroEL are consistently elevated in LAB exposed to milk lipids or MFGM fractions [180,193]. Their induction reflects misfolding of membrane-integrated proteins during fluidity shifts.

Oxidative stress pathways play a key role because PUFA oxidation generates ROS, lipid peroxides, and aldehydes. LAB respond by inducing methionine sulfoxide reductase and thioredoxin/thioredoxin-reductase systems [103,193,194]. DNA repair systems, including RecA and UvrABC endonucleases, are activated, consistent with oxidative lesions and membrane-derived stress signals [174]. The magnitude and scope of this coordinated stress response differ markedly between LAB populations. Native strains mount a broader transcriptional and proteomic stress signature, displaying more chaperone variants, multiple redox enzymes, and a wider stress-associated regulon. This reflects their evolutionary exposure to fluctuating lipid profile in milk, contrasting with the narrowed stress repertoire of domesticated starter LAB [186].

##### Cholesterol and Minor Lipid Fractions

Cholesterol contributes to membrane order by stiffening the bilayer, reducing permeability, and stabilizing sphingomyelin-rich microdomains. Through these physical effects, cholesterol modulates membrane protein insertion, the activity of membrane-associated enzymes, and the performance of substrate transport systems. Supplementation with cholesterol has been shown to shift the fatty-acid composition of LAB membranes [105] and to alter their proteome toward enhanced envelope stability.

Minor milk lipid fractions include mono- and diacylglycerols, ceramides, and glycolipids. Mono- and diglycerides influence membrane curvature and protein clustering, while ceramides and glycosphingolipids contribute to membrane rigidity and microdomain formation and can modulate bacterial adhesion to host epithelial cells [178,195]. Glycolipids may serve both as carbon sources and as signaling molecules, particularly for native species that possess broader catabolic capabilities [186]. When these lipids associate with bacterial membranes, they influence hydrophobic interactions, reinforce membrane ordering, and alter surface protein exposure patterns in manners that can enhance or diminish adhesion to surfaces or host tissues. Such modifications have significant implications for biofilm formation, interbacterial aggregation, and immune-modulatory interactions, as the distribution of surface proteins determines the recognition profile and interaction strength with host pattern recognition receptors. These lipid-driven changes can therefore fundamentally reshape the ecological fitness of bacteria, promoting survival advantages under certain milk-processing conditions or within the gastrointestinal environment. Taken together, milk lipids remodel bacterial membranes, reprogram proteomic profiles, alter metabolic fluxes, modulate stress responses, and shape ecological and host-interaction outcomes [95,177,187].

#### 3.2.4. Functional Consequences of Lipid-Induced Remodeling

The intricate molecular adaptations—membrane remodeling, metabolic rewiring, and stress response activation—translate directly into significant functional phenotypes that determine bacterial ecology and utility in food systems. One of the most critical observable phenotypes is enhanced acid tolerance. By stabilizing the membrane structure (reducing proton permeability) and upregulating the F_0_F_1_-ATPase system, lipid-adapted cells more effectively maintain the ΔpH and sustain intracellular pH homeostasis, enabling increased viability under acidification stress [186]. Similarly, cryotolerance is improved, as MFGM phospholipids protect membranes during freeze–thaw cycles by preventing low-temperature phase transitions that would otherwise damage the bilayer and membrane-associated enzymes [186,193].

Beyond survival, functional traits related to the external environment are significantly modulated. Adhesion and colonization capabilities are altered as lipid-modified membranes enable the rearrangement of surface proteins. This influences binding to milk matrix components (like casein micelles) and to biological surfaces (like intestinal epithelium and mucus). These lipid-driven interactions enhance adhesion capacity and are relevant for probiotic functionality and application potential [174,185].

Furthermore, lipid-induced metabolic reprogramming directly impacts the production of flavor and aroma compounds. The enhanced metabolic diversity and redirection of Acetyl-CoA flux result in elevated production of volatile compounds, including aldehydes, ketones, alcohols, and acetoin [196]. These shifts in volatile profiles contribute to more complex sensory profiles in fermented dairy products. Finally, the superior metabolic breadth and enhanced adhesion and stress-management capabilities observed in native milk bacteria enable them to competitively dominate microbial communities under complex lipid exposure, thereby influencing microbial succession and the ecological outcome during fermentation [174].

## 4. Discussion

The current study provides a comprehensive mechanistic overview of how extrinsic and intrinsic factors indirectly shape the cellular physiology of LAB and IMM through the modulation of the MLP. Our analysis confirms that membrane remodeling, triggered by the assimilation of these modified milk lipids, constitutes the central adaptive axis regulating bacterial homeostasis in the milk environment. The most notable finding of this work is the demonstration that IMM exhibit a significantly broader proteomic and adaptive plasticity—particularly in the regulation of the FASII pathway and compensatory stress responses like the F_0_F_1_-ATPase complex—when compared to domesticated starter cultures. This differential capacity for membrane fluidity compensation and metabolic restructuring highlights a profound ecological and evolutionary distinction. The enhanced adaptive breadth of native microorganisms, stemming from continuous exposure to lipid cues whose composition is dynamically influenced by factors such as the animal’s diet (extrinsic) and lactation stage (intrinsic), suggests a specialization that provides a measurable competitive advantage over the industrially adapted starter strains. This insight is important for understanding the complex biophysical and metabolic interactions governing the stability and functionality of dairy fermentation systems.

### 4.1. Theoretical Implications

The collective evidence firmly establishes the multifactorial regulation of the MLP, which is dynamically governed by both intrinsic factors (breed, genetics, lactation stage) [5,66,68,70,71,75] and extrinsic factors (dietary interventions, environment). Genetic polymorphisms in key enzymes, such as DGAT1 and SCD1, reinforce the genetic basis for FA variability [35,66,68]. The dynamic shifts observed across lactation and following dietary lipid supplementation underscore the significant physiological plasticity of the mammary glands’ lipid metabolism [28,31,32,82,197].

The critical role of RH is highlighted [72], not only as the primary driver of FA profile changes but also through its intermediates, like trans-10, cis-12 CLA, which provide mechanistic insights into phenomena such as MFD [61,62,198]. The endogenous synthesis of CLA in the mammary gland from vaccenic acid further challenges simplistic views of dietary FA transfer [120,153,199]. Moreover, the novel view of milk lipids as modulators of bacterial protein expression in dairy matrices introduces a new functional dimension, suggesting an influence on fermentation processes [26,27,200]. The observed effects of bioactive FAs, including odd- and branched-chain FA, question long-standing assumptions that saturated fats are uniformly detrimental to human health [94,201]. Comparative lipidomic analyses reveal breed-specific lipidomic signatures [23,25,73], and the integration of advanced omics methodologies (UHPLC-MS/MS, RNA-Seq) has been pivotal in refining these theoretical models [67,202].

### 4.2. Practical Implications

The theoretical understanding has direct implications for dairy management strategies. The proven efficacy of nutritional management, particularly through the use of pasture-based feeding [17,203] and supplementation with oilseeds, marine lipids, and agro-industrial by-products [1,18,34,39,41,46,91,92], provides an efficient tool for enhancing the concentration of health-promoting FA (n-3 PUFA, CLA) in milk, supporting sustainable production practices and meeting consumer demand [143,204,205]. Complementing diet, genetic selection programs targeting favorable FA polymorphisms offer a long-term, cumulative avenue for improving milk fat composition at the herd level [5,35,66,68,69,70].

Furthermore, understanding the variations across lactation stage and breed allows for tailored management practices that optimize milk quality and consistency for processing [22,28,31,83]. The insight into the lipid-microbe interaction offers the potential for developing novel dairy biotechnologies through deliberate manipulation of microbial fermentation [26,27], which can lead to value-added products creation, and market oriented innovation [206,207] while maintaining sensory qualities [82,208]. This type of innovation, due to its specificity close to personalization, may offer potential benefits for small and medium-sized dairy enterprises [207]. The necessity for improved rumen-protection technologies remains evident to maximize the transfer efficiency of beneficial FA, as current methods demonstrate limitations [72]. Finally, the application of rapid analytical techniques (MIR advanced MS) facilitates precision dairy farming and routine quality control [5,202], supporting public health policies aimed at enhancing the nutritional value of dairy products [19,209]. Lipid-responsive regulation of bacterial protein expression is technologically relevant and a powerful tool. Recent work on *Lacticaseibacillus paracasei* Shirota demonstrates that this species can produce a diverse set of bioactive metabolites—including bioemulsifiers, lipase, bacteriocins and antidiabetic compounds, whose synthesis is strongly influenced by medium composition and pH, highlighting its metabolic plasticity in lipid-rich environments [192]. Such findings are particularly relevant for artisanal dairy fermentations, where complex microbial communities, including occasional pathogens and metabolically versatile LAB, respond to milk lipid composition through coordinated proteomic and metabolic adjustments.

### 4.3. Agreement and Divergence Across Studies

The reviewed literature shows consensus that the milk FA profile is significantly and effectively influenced by dietary interventions, such as supplementation with oilseeds, plant oils, and marine organisms’ origin. This complex landscape of agreement and divergence is further analyzed in Table 3.

These modifications reliably improve the nutritional quality of milk by increasing beneficial UFA, including CLA, omega-3 PUFA, and MUFA, while simultaneously reducing saturated fats. There is also strong agreement on the crucial role of RH in mediating these dietary effects, with processing methods (like using protected fats) significantly influencing the final outcome. Intrinsic factors, namely breed, genetics, and lactation stage, are consistently acknowledged as influencing milk lipid composition, although their effect size is generally reported as moderate compared to dietary inputs.

However, several areas of divergence remain in the literature. The precise degree to which breed and specific genetic markers dictate the overall lipid variation is debated. The magnitude of changes in milk yield resulting from lipid supplementation and the comparative efficacy of specific lipid sources, show inconsistencies across studies. The long-term health impacts of consuming modified milk fat are areas lacking full consensus. The current boundaries of knowledge regarding how milk lipids shape bacterial protein expression in dairy fermentation are still incomplete; however, the findings synthesized in this review clarify what is already established and delineate the specific gaps that ongoing research aims to address. The inconsistencies are largely attributed to methodological variances (in analytical techniques and study designs), as well as species-specific and environmental factors [5,75,84,210].

**Table 3:** Agreement and Divergence Across Studies.

Comparison Criterion	Studies in Agreement	Studies in Divergence	Potential Explanations
Dietary influence	Most studies concur that supplementation with oilseeds (e.g., linseed, soybean), plant oils, marine lipids, and agro-industrial by-products successfully modify milk fatty acid (FA) profiles, enriching milk with conjugated linoleic acid (CLA), omega-3 PUFA, MUFA [45,47,91,92,117]. Protected fats and processing methods (extruded, formaldehyde-treated) also influence transfer efficiency and RH [119,211]. Similarly, multiple studies agree that pasture-based diets and supplementation with PUFA (e.g., flaxseed, rapeseed) increase beneficial UFA in milk and improve lipid profiles [1,33,34,43]. Specifically, feeding alfalfa silage or flaxseed enhances omega-3 PUFA content [33,39]. Conversely, diets rich in SFA increase milk fat production but reduce de novo FA synthesis [34].	Some studies report limited or variable effects of diet on milk lipid profiles, especially depending on breed and lactation stage [4,28]. Inconsistent effects of specific lipid sources or supplementation levels on milk yield and fat content are also reported; for instance, the effects on milk yield and MFD vary with the type and amount of supplementary lipids [36,40], sometimes reducing milk fat but not yield, as seen with sunflower oil [47,91]. Furthermore, the impact of by-products like pumpkin or hempseed varies and requires further research [45].	Variability in study outcomes regarding the effect of diet on milk composition is explained by multiple factors, including differences in lipid source types, levels of inclusion, cow breed, and lactation stage. Other key variables include experimental design (e.g., in vivo studies vs. meta-analysis), processing methods, and animal factors, which further modulate responses. Additionally, feed types (e.g., pasture vs. silage), study duration, and the overall diet formulation contribute to the observed variation.
Milk FA composition	Consensus exists that dietary supplementation increases beneficial FA like CLA, vaccenic acid, oleic acid, and omega-3 PUFA while decreasing SFA, including palmitic acid (C16:0) [34,37,82,91,92,117]. Feeding fresh pasture or grazing enhances milk CLA and omega-3 PUFA content relative to total mixed rations [17,89,90,203,212,213].	The degree to which milk fat content and overall yield are affected diverges. Some meta-analyses indicate no effect on milk yield but reduced fat content [34,91]; others report variable MFD linked to specific FA isomers or lipid types [61,62]. Reports differ on the exact changes in minor FA and health-relevant isomers.	Variations stem from differences in animal breed, diet composition, rumen microbial populations, and study duration. The form of fat supplement (protected vs. unprotected) and RH patterns affect milk FA outcomes.
Genetic variation	Breed and genetic polymorphisms (e.g., DGAT1, SCD1, Protein acidic enriched protein) significantly affect milk FA composition and lipid synthesis pathways [5,23,24,35,66,75] with moderate effects, including on CLA and SFA proportions [68,69,70,71].	Some studies find minor breed differences or low genetic variation impact, especially in native or dual-purpose breeds vs. mainstream breeds [5,23,71,72]. Differences in genomic markers’ effects across breeds are observed [5,35].	Divergence in research findings is often attributed to the genetic architecture differences among breeds, including the fixation of specific polymorphisms (e.g., DGAT1 in Norwegian Red cattle). Further disparities arise from variations in the breeds studied, the sample sizes, the specific genetic markers assessed, and the resulting environmental-genetic interactions observed across studies.
Environmental and physiological effects	The lactation stage significantly influences milk FAs profiles, with changes in FA proportions reported across species during early, mid, and late lactation [1,22,28,73]. This effect is often related to the cow’s energy balance and metabolic status, with early lactation commonly linked to increased mobilization of SFA from body reserves [63,72]. Furthermore, environmental conditions and animal welfare status are also reported to modulate the overall milk lipid composition [2,30].	Some studies note breed-specific responses to environmental factors; local breeds show healthier profiles under similar conditions compared to cosmopolitan breeds [44,73]. The magnitude of lactation stage effects varies [1,22].	Different breeds and species respond variably to environmental stimuli; management practices and measurement timing during lactation influence outcomes.
RH modulation	Studies agree that RH extensively saturates dietary UFA, limiting their direct transfer to milk. Strategies using protected fats, processing (e.g., extrusion, formaldehyde treatment), and inclusion of marine lipids or plant secondary compounds modulate RH pathways to increase UFA outflow [72,91,93,119,120]. The role of rumenbacteria (e.g., *Butyrivibrio*) and protozoa in RH and FA protection is recognized [72].	The extent to which different supplements inhibit RH or how rumen microbiota shifts affect milk FA varies. Some supplements (e.g., fish oil) can impair rumen fermentation and decrease feed intake [208], while others show inconsistent effects on RH inhibition [93].	Differences in diet basal composition, dose, duration, and ruminal environment contribute. Species differences (cow vs. goat/sheep) and microbial community variability also explain inconsistencies. Protective technologies vary in effectiveness.
Health-related lipid outcomes	There is agreement that modifying milk fat to increase UFA, including CLA and omega-3 PUFA, holds potential to improve human health by reducing cardiovascular risk and providing bioactive lipids [19,40,72,82,117,121,143,209]. Milk fat’s complex matrix and minor components like dairy phospholipids add beneficial effects [204].	Some debate remains on the cardiovascular impact of dairy SFA and trans fats, with recent studies questioning the negative associations and highlighting the importance of the food matrix [94,205]. The long-term effects of altered milk FA profiles on human health remain inconclusive [19,188,209].	Variability due to study design (epidemiological vs. intervention), population heterogeneity, and incomplete understanding of dairy fat metabolism in humans. Differences in milk FA profile modifications and consumption patterns also contribute.
Analytical methodologies	Gas chromatography (GC), mass spectrometry (MS), liquid chromatography (LC), and mid-infrared spectroscopy (MIRS) are widely employed to profile milk lipids and FAs [1,5,23,33,124]. Combining untargeted proteomics and lipidomics provides insights into milk composition and bioactivity [31,67].	Accuracy and predictive power of analytical methods vary by FA type, breed, and sample matrix; for example, MIRS predictions show biases for some FA [5,75,87]. Different lipid extraction and fractionation methods lead to variation in results [210].	Variations in sample preparation, instrumentation sensitivity, calibration models, and breed-specific milk composition affect analytical outcomes and comparability across studies.

### 4.4. Limitations of the Literature Data

The limitations of the literature are primarily defined by the inconsistent and variable effects reported across studies, which can be attributed to a combination of experimental design, dietary factors, and intrinsic animal characteristics (Table S3). Divergence in research findings is largely driven by inconsistency and variability in study design, leading to reports of limited or variable effects of diet on MLP [4,28,211]. The effects on milk yield and MFD vary considerably with the type and amount of supplementary lipids, sometimes resulting in inconsistent outcomes, such as reduced milk fat content without a concurrent reduction in milk yield [36,40,47,91]. Furthermore, the specific impact of certain by-products (e.g., pumpkin or hempseed) remains variable, suggesting a need for further research [45]. This extensive variation is explained by a multitude of modulating factors. The dietary factors, differences in the experimental design (e.g., in vivo studies vs. meta-analysis), study duration, feed types (e.g., pasture vs. silage), and overall diet formulation are major contributors to result variability [205,210,212,213]. Responses are also strongly influenced by feed supplements processing methods (e.g., extruded, formaldehyde-treated) and the specific type and level of lipid inclusion [4,28]. Intrinsic and environmental factors are also critical modulators of responses with the impact on fat milk composition. Lactation stage significantly influences FA profiles across species, often linked to changes in energy balance and metabolic status [1,22,28,63,72,73]. Genetic architecture differences among breeds are central to divergence, driven by variations in breeds studied, sample sizes, specific genetic markers assessed, and the fixation of polymorphisms (e.g., DGAT1 in Norwegian Red cattle), leading to distinct environmental-genetic interactions [4,28]. Finally, environmental conditions and animal welfare status are also reported to modulate the milk lipid composition and is responsible for the inconsistent and variable effects reported across studies [2,30].

### 4.5. Gaps and Future Research Directions in Milk Lipid Manipulation for Microbial Protein Modulation

Future research efforts aimed at manipulating MLP must address significant mechanistic, methodological, and translational gaps to achieve consistent, sustainable, and validated milk quality enhancement (Table S4).

A key research objective is to elucidate the mechanistic links between milk lipids and bacterial protein expression, as limited direct evidence exists on how specific MLP modulate microbial function in dairy applications [26,27]. This requires conducting integrative in vitro and in vivo multi-omics studies, combining milk lipidomics with bacterial transcriptomics and proteomics to unravel the complex, multifactorial interactions that influence dairy product quality [30,36,205,210]. Furthermore, there is a recognized need to develop and validate standardized protocols for lipid extraction and multi-omics analyses to ensure comparability and reproducibility across studies, which is essential for building a coherent knowledge base [1,202].

The variability and complexity of RH remain a major challenge, limiting the consistent enhancement of UFA in milk [32,93]. Future studies should focus on conducting longitudinal and mechanistic studies to characterize rumen microbial populations and their RH pathways, particularly investigating the species-specific roles of rumen protozoa in UFA protection and intermediate production [72]. This microbial understanding should be complemented by technological innovation, specifically by testing novel rumen-protection technologies that improve the transfer efficiency of PUFA into milk fat without reducing intestinal digestibility.

Genetic and physiological variability also necessitate focused research. Genetic studies often rely on a limited number of breeds, leading to poor generalizability [66]. Research must expand genomic and transcriptomic analyses to diverse breeds and develop combined breeding and feeding programs that consider gene-diet interactions using [68,70]. Similarly, most studies assess lactation stage effects cross-sectionally, requiring the design of longitudinal cohort studies to track the temporal dynamics of milk lipids and their functional roles throughout an individual animal’s lactation [1,28]. These efforts should be complemented by controlled experiments that quantify environmental variables and animal welfare indicators to better understand their modulating effects on milk composition [2].

Finally, translational research remains a critical gap. Current human clinical evidence remains limited to validate the health benefits of consuming milk with altered FA profiles [19,94,143]. Designing and implementing properly planned randomized controlled trials (RCTs) is crucial [121]. Concurrently, efforts should focus on functional validation through targeted in vitro assays using isolated milk lipid fractions to assess their biological effects on microbial and intestinal cells. Additionally, the field needs to address commercial limitations by exploring dose–response relationships for dietary supplements to minimize MFD and maintain sensory quality [47,82]. Future research should also expand to include MFGM phospholipids, which exhibit considerable nutraceutical potential but are currently understudied compared to fatty acids [204].

## 5. Conclusions

This comprehensive review successfully synthesizes two traditionally separate fields—livestock nutritional management and microbial proteomics—to establish a clear, integrative framework. We confirmed that the MLP is a highly plastic and modifiable trait, shaped by a complex interplay of genetic, dietary, and environmental factors. This review establishes that lipids act not only as nutrients, but as bioactive environmental signals that significantly influence the functional state of both IMM and starter culture bacteria. The evidence compiled highlights the direct connection between lipid composition and the modulation of structural and enzymatic proteins, including the emerging role of moonlighting proteins, which allow bacteria to adapt efficiently to changes in the dairy matrix.

The central finding of this narration is the identification of a significant knowledge gap: a substantial lack of studies employing an Omics-integration approach to link MLP modulation directly to microbial proteomic outcomes in dairy systems. We propose that exploiting this lipid-proteome axis represents a promising conceptual framework for the dairy industry. Future research must focus on in vivo and ex vivo proteomic analyses using milk from manipulated diets and farming styles to move beyond correlational data. By actively managing the cow’s lipid output, the industry can strategically enhance dairy product safety, flavor development, and nutritional functionality, positioning milk fat as a functional biological determinant within dairy systems.

## Figures and Tables

**Table 1:** Direct Influence of Intrinsic and Extrinsic Factors on Milk Lipid Profiles.

Theme	Appears In Papers	Description
Dietary influence on milk lipid profile	63/138	Diet is confirmed as the primary modifiable factor influencing milk FA profiles. Strategies focus on altering diet composition—including pasture type (multispecies swards), forage preservation (silage vs. hay), and extensive lipid supplementation. Supplementation involves vegetable oils (linseed, soybean, sunflower, rapeseed), marine lipids (fish oil), and protected fats. These approaches significantly alter FA composition by affecting de novo synthesis and milk fat yield, leading to enrichment in beneficial FAs, such as n-3 PUFAs, CLA, and MUFAs. The modifications enhance the milk’s nutritional and techno-functional properties, typically without compromising milk yield [1,2,4,14,19,21,22,27,28,29,30,31,32,33,34,35,36,37,38,39,40,41,42,43,44,45,46,47,48,49,50,51,52,53,54,55,56].
Use of alternative feed sources, protected fats, by-products	26/138	Incorporation of rumen-protected fats, oilseed industry by-products, and unconventional feeds (e.g., grape, pomegranate, olive by-products, microalgae) is effective in enriching milk with unsaturated fatty acids (UFA) without negatively affecting milk production. These strategies also offer sustainability benefits by utilizing agro-industrial residues and reducing feed cost [26,27,28,33,39,43,45,46,47,48,49,53,57,58,59,60].
Regulation and metabolic effects on milk fat synthesis	14/138	Studies focus on the molecular regulation of milk fat synthesis, including the effects of specific FAs like trans-10, cis-12 CLA in inhibiting milk fat production. The metabolic consequences of milk FA on energy balance, inflammation, and lipid metabolism in dairy cows and humans are also examined [18,34,36,37,61,62,63,64,65].
Genetics’ influence on milk fatty acid composition	51/138	Genetic factors, including breed differences (e.g., indigenous vs. cosmopolitan breeds) and polymorphisms in key enzymes such as stearoyl-CoA desaturase (SCD/SCD1), DGAT1, Protein acidic enriched protein, and Nuclear receptor coactivator 6 (NCOA6), are primary drivers of milk FA variability. Also, the co-occurrence of some fat parameters with casein genetic variants is noted. The genetic makeup influences de novo FA synthesis and the relative abundance of saturated vs. UFA. Breed-specific lipidomic and proteomic variations determine the functional and health-promoting properties of the milk. Utilizing genetic selection in combination with nutritional strategies offers a permanent method for long-term enhancement of milk fat quality [1,2,4,5,18,19,20,21,22,35,36,37,38,39,44,64,65,66,67,68,69,70,71,72,73,74,75,76].
Epigenetic influence on milk FA composition	6/138	Epigenetic mechanisms (DNA methylation, histone modifications, and miRNAs) serve as molecular switches, providing the link between a dairy animal’s metabolic state and diet and the actual FA profile secreted into the milk. These nutrient-responsive processes regulate the accessibility and expression of key lipogenic genes (e.g., acetyl-CoA carboxylase, FASN, SCD), where modifications can either suppress *de novo* FA synthesis (e.g., via methylation) or activate it (e.g., via ω−3-promoted histone acetylation) to influence the final composition of milk fats [65,77,78,79,80,81].
Comparative analysis across milk sources and species	14/138	Comparative studies highlight differences in lipid profiles among milk from cows, goats, sheep, and buffaloes, reflecting species-specific metabolic pathways and feeding practices. Such differences extend to TAG composition and lipid quality indices, with implications for product development and nutritional value. Cross-species analyses underscore the importance of tailored breeding and feeding strategies [21,36,44,52,56,72,74,81,82,83,84,85,86,87].
Environmental and welfare factors influencing milk lipids	21/138	The animal’s physiological status and stage of lactation are essential modulators of milk FA composition. Early lactation, marked by body fat mobilization, shifts the profile toward decreased de novo FAs and increased long-chain FAs, whereas late lactation often shows an increase in saturated FAs. Metabolic health issues like ketosis and overall energy balance also substantially affect milk fat synthesis. Separately, environmental conditions, animal welfare, and management practices indirectly influence lipid profiles. Factors like seasonal variations and housing systems alter feed quality, animal health, and stress levels. The synergy of these physiological and environmental changes affects milk yield, metabolic stability, and the final nutritional/functional value of the milk, impacting the sustainability of dairy production [1,2,18,24,26,27,74,82,88,89,90].
Ruminal biohydrogenation (RH) and microbial metabolism impact on milk lipid composition	40/138	The complex ruminal processes of lipolysis and RH substantially influence the saturation level and isomer profile of milk FAs. Research highlights the role of specific rumen bacteria and protozoa in converting UFA into saturated forms and bioactive intermediates like vaccenic acid and CLA. Manipulation of rumen microbial populations and RH pathways through diet or additives such as tannins and essential oils can improve the milk FA profile [13,17,30,31,32,33,35,38,52,71,82,84,85,86,91,92,93,94,95,96,97,98,99,100,101,102,103,104,105,106,107,108,109,110,111,112,113,114,115,116].
Nutritional, health and technological implications	94/138	Processing technologies and biological interventions are actively explored to enhance both the healthfulness and the functional attributes of milk fat for diverse dairy products. Milk enriched with beneficial FAs, such as PUFAs and CLA, confers health advantages, including anti-inflammatory and cardioprotective effects. Alterations in milk fat composition not only affect nutritional quality but also critical technological properties like spreadability and cheese-making traits. Furthermore, these lipids influence the organoleptic properties and consumer acceptance of dairy products. Research is increasingly urging a nuanced understanding of milk fat’s effects within the dairy food matrix, moving beyond simple saturated fat content, to fully grasp its public health relevance and technological utility [1,13,14,15,16,17,30,31,32,33,35,40,41,45,46,47,50,55,60,62,65,66,68,69,70,71,81,84,85,86,87,88,89,90,91,92,93,94,95,96,97,98,99,100,101,102,103,104,105,106,107,108,109,110,111,112,113,114,117,118,119,120,121,122,123,124,125,126,127,128,129,130,131,132,133,134,135,136,137,138,139,140,141,142,143].

**Table 2:** Bacterial strains, lipid types, and mechanistic impacts.

Taxonomy	Native/Starter	Lipid Type Tested	Proteomics (Observed)	Membrane Remodeling	Metabolism	Stress Response	Adhesion	Fermentation Phenotype	Ref.
*Lacticaseibacillus casei* Shirota; formerly *Lactobacillus casei* Shirota *	Starter/probiotic	PUFA (linoleic, arachidonic)	↑ Growth-linked proteins at low PUFA levels; ↓ protein abundance at high PUFA levels	Incorporates PUFAs → ↑ membrane fluidity	Low PUFA ↑ growth; high PUFA ↓ FAS pathways	Mixed: low PUFA ↑ tolerance; high PUFA causes stress	↑ at low PUFA ↓ at high	Not reported	[127,175]
*Limosilactobacillus reuteri* DSM 17938; formerly *Lactobacillus reuteri* DSM 17938	Native/probiotic	Breast-milk fatty acids	↑ Pyruvate dehydrogenase, ↑ enolase, ↑ amino-acid enzymes	FA incorporation into membrane	↑ Central carbon flux, ↑ Antioxidant precursors	↑ Antioxidant/stress tolerance	Not reported	↑ Survival/growth in milk	[102]
*Lactiplantibacillus plantarum* (various strains); formerly Lactobacillus plantarum *	Starter	FAs: oleic, linoleic, Tween 80	↓ FASII proteins; ↑ stress-tolerance proteins	↑ Cyclopropane FA; membrane stiffening	↓ De novo FA synthesis; ↑ FA incorporation	↑ Tolerance to drying/high pressure (HHP)	Surface changes; adhesion-modifying	Improved industrial survival	[103,188]
*Lacticaseibacillus gasseri* (strain not specified); formerly Lactobacillus gasseri *	Native	Trans FAs	↑ Metabolic proteins; ↑ growth-linked proteome	↑ Cellular lipid content	↑ Metabolic activity	Not highlighted	Not reported	↑ Growth	[129]
*Fructilactobacillus sanfranciscensis* ATCC 27651; formerly *Lactobacillus sanfranciscensis* ATCC 27651 *	Starter	PUFA (oleic, linoleic)	↓ Growth-related proteins (PUFA sensitivity)	Membrane FA incorporation; ↑ susceptibility	↓ Growth; inhibition by PUFAs	↑ Stress due to PUFA exposure	Altered surface properties	↓ Fermentation performance	[103]
*Lactobacillus delbrueckii* subsp. *bulgaricus*	Starter	PUFA; endogenous PL classes	↑ Glycolysis proteins; ↑ translation factors	Lipid profile variation (cardiolipin ↑ tolerance)	↑ FA biosynthesis during acid adaptation	↑ Acid-stress proteins	Not reported	Differences in drying survival	[189]
*Lactobacillus rhamnosus* GG	Starter/ porbiotic	Whey based media vs. MRS	↑ Glycolysis proteins; ↑ transcription/translation factors	Membrane FA incorporation; ↑ ABC transporters	↓ FA biosynthetic genes; ↓ FabZ, FabF and FabK, CFAS	↑ Acid-stress tolerance; ↑ Shock protein production, growth phase related;	Surface changes; adhesion-modifying	Shift glucose to galactose utilization; the transition from homolactic to mixed acid fermentation	[171,172]
*Escherichia coli* (lab strains)	Native	Long-chain exogenous FA	↓ FASII proteins (FabA/B/G); ↑ FA-uptake proteins	Acyl-ACP remodeling; ↑ exogenous FA incorporation	↓ De novo FA synthesis; metabolic rerouting	↑ Tolerance to drying/high pressure. Envelope stress activation	Not reported	Not a fermentation organism	[177]
*Enterococcus faecalis* mprF−	Native/pathogen	Loss of L-PG; palmitic/stearic FA rescue	↓ Secretion proteins; ↓ FA synthesis proteins	Massive PL remodeling; ↑ long-chain acyl-ACP	↓ Endogenous FA synthesis; ↑ exogenous FA rescue	↑ Biofilm-linked stress pathways	↑ Biofilm	Pathogenic (not fermentation)	[95,179]
*Bacillus subtilis* (wild type)	Native	Exogenous FA incorporation	↓ Flotillin; ↓ MreB; ↑ FA enzyme remodeling	Altered membrane domains	↑ FA degradation enzyme changes	Altered stress capacity	Not reported	Not a fermentation organism	[190]
*Pseudomonas aeruginosa ΔPlaF*	Pathogen	PL acyl-chain alteration	↑ Proteases, ↑ iron-uptake; ↑ TCS regulation	Altered PL acyl-chains	Metabolic secretion changes	↑ Biofilm regulatory stress	↑ Biofilm potential	Pathogenic (not fermentation)	[191]
*Streptococcus agalactiae*	Pathogen	Serum FAs	↓ FASII proteins (3–40×); ↑ FA-uptake	↑ Exogenous FA incorporation	↓ Endogenous FASII; ↑ metabolic bypass	Virulence stress preserved	Not reported	Virulence maintained	[130]
*Lacticaseibacillus casei* Shirota; formerly *Lactobacillus casei* Shirota *	Starter/probiotic	Endogenous glycerophospholipids	↑/↓ survival-linked proteins; PL-correlated proteome	Distinct PL species predict tolerance	Metabolic pathways tied to PL composition	↑ or ↓ tolerance to processing	Not reported	Strongly strain-dependent survival	[192]

* former taxonomic names; ↑ increased effect; ↓ decreased effect; → is related to.

## Data Availability

No new data were created or analyzed in this study. Data sharing is not applicable to this article.

## Supplementary Materials

The following supporting information can be downloaded at: https://www.mdpi.com/article/10.3390/ani16030477/s1, Table S1: Intrinsic and Extrinsic Factors impact on Milk Lipid Profiles; Table S2: Intrinsic and Extrinsic Factors impact including Rumen Biohydrogenation on Fatty Acids composition; Table S3: Limitations of the Literature; Table S4: Gaps and Future Research Directions. References [49,50,51,52,53,54,55,56,58,59,60,87,115,116,139,140,141,142,143,144,145,152] are cited in the Supplementary Materials.

## Abbreviations

ABC—ATP-Binding cassette; CLA—Conjugated linoleic acid; DGAT1—Diacylglycerol O-acyltransferase 1; DnaK–DnaJ–GrpE—Chaperone complex; FA—Fatty acid; FabA, FabB, FabG, FabD—Enzymes of the fatty acid synthesis II pathway; FASN—Fatty acid synthase; F_0_F_1_-ATPase—F_0_F_1_-Adenosine triphosphatase; FFA—Free fatty acid; GroEL/GroES—Chaperone complex; IMM—Indigenous milk microbiota; LAB—Lactic acid bacteria; LCFA—Long-chain fatty acid; MCFA—Medium-chain fatty acid; MFD—Milk fat depression; MFGs—Milk fat globules; MUFA—Monounsaturated fatty acid; PUFA—Polyunsaturated fatty acid; RH—Rumen biohydrogenation; SCFA—Short-chain fatty acid; SCD/SCD1—Stearoyl-CoA desaturase; SFA—Saturated fatty acid; TAG—Triacylglycerols; UFA—Unsaturated fatty acids.
