# Supplementary material for: Milk Lipids as Bioactive Modulators of the Bacterial Proteome: Mechanisms Linking Dairy Management to Microbial Performance
Source: Animals (Basel). 2026 Feb 3;16(3):477. doi: 10.3390/ani16030477 (PMC12896685; doi:10.3390/ani16030477)
Supplement: Supplementary file 1 [file animals-16-00477-s001.zip › animals-4071760-supplementary.pdf]

No.	Reference	Dietary Influence	Genetic Variation	Environmental and Physiological Effects	Analytical Methodologies
1	[1]	Pasture diet alters PUFA and TAG profiles across lactation stages	Breed influences FA profile; Holstein-Friesian *vs* Jersey crossbreed differences	Lactation stage strongly affects TAG and polar lipids	GC-FID, HPLC-ELS-MS,multivariate analysis
2	[2]	Screening of intrinsic and extrinsic factors affecting cow and goat milk liposoluble components	Breed effects limited but present; species differences notable	Lactation stage effects linked to body fat mobilization	Multivariate factor analysis
3	[3]	Feeding ration and lactation month majorly affect nutritionally important FA profile	Breed and parity have minor effects on FA composition	Seasonal and lactation effects quantified via R2 values	Statistical modeling of FA composition
4	[5]	Not primary focus	Genetic variation in milk FA composition among Dutch cattle breeds Breed differences minor; Holstein shows more genetic variation	Lactation and environment influence FA profiles	MIR spectrometry, GC-MS
5	[5]	Dietary C18 PUFA effects on rumen microbiota and milk CLA	Dietary fat modulates rumen fermentation and milk FA	Environmental and microbial interactions studied; Milk CLA linked to rumen microbial activity	Multivariate factor analysis
6	[6]	Not primary focus	Breed differences noted within lactation stages	Lactation stage major determinant of FA and lipid indices in sheep	FA profiling, lipid quality indices
7	[7]	Breed-specific fat and protein content differences linked to diet	Significant breed differences in FA and peptide profiles	Health status inferred from conductivity; breed effects on digestion	Lactoscan, MALDI-TOF, GC-MS, *in vitro* digestion
8	[8]	Not primary focus	Genetic variation in milk fat composition and breeding implications	Not focused on environmental or physiological factors	Genetic parameter analysis
9	[9]	Seasonal diet variation affects TAG species in buffalo and cow milk	Breed and season strongly influence TAG profiles	Seasonal variation more pronounced in Holstein than buffalo	MS/MS spectral analysis

No.	Reference	Dietary Influence	Genetic Variation	Environmental and Physiological Effects	Analytical Methodologies
10	[10]	Linseed diet induces milk fat depression; gene expression varies	SNPs in 15 key genes regulate milk fat synthesis sensitivity; Milk fat de-pression linked to gene regula-tion	Diet-induced gene expression changes during lactation	RNA-seq, SNP discovery
11	[11]	Marine lipid supplementation affects milk fat synthesis genes in goats	Gene networks differ between responsive and non-responsive goats; lipids modulate gene expression in mammary cells	Not focused on environmental or physiological factors	RNA-seq, WGCNA
12	[12]	Effects of ration; Corn silage increases SFA; pasture increases PUFA; Effects of ration.	Not primary focus	Lactation phase, season and parity significantly affect FA profiles	Mixed models, GC-MS
13	[13]	Not diet-focused;	Not gene-focused; Milk FA reflects energy metabolism and liver health in Holstein cows	Early lactation metabolic indicators correlated; Milk FA profile associations with metabolic status	Multivariate factor analysis
14	[14]	PUFA supplementation effects on skimmed milk proteome and affect mammary lipogenesis process	Not primary focus	Early lactation dynamics studied lactation day modulate milk ingredients profiles	MS quantitative analysis
15	[15]	Alfalfa silage increases ω-3PUFA and phospholipids in milk	Single breed (Holstein) studied; no genetic variation focus	Roughage type impacts lipidomic profile	UHPLC-MS/MS, GC-MS
16	[16]	Flaxseed and rape seed feeding effects on milk FA in crossbred cows; Oilseed supplementation increases unsaturated FA, decreases SFA	Not primary focus	Not primary focus	GC analysis
17	[17]	Meta-analysis of dietary FA profile effects on milk fat production	Not primary focus	Diet and animal production traits predict milk FA profile	Meta-regression analysis

No.	Reference	Dietary Influence	Genetic Variation	Environmental and Physiological Effects	Analytical Methodologies
18	[18]	Agro-industrial by-products improve milk FA profile without yield loss	Not primary focus	Environmental sustainability considered; By-products modulate rumen biohydrogenation and milk FA	Review offering trials
19	[19]	Diet combined with breed affects sheep milk FA profile	Breed significantly affects FA groups and metabolic pathways	Regional farming system influences FA composition	Multivariate factor analysis, ANOVA
20	[20]	Strategic supplementation and pasture diet modulate milk FA via genotypes	DGAT1 and SCD1 genotypes strongly influence milk FA profile	Not primary focus	Genotyping, milk FA profiling
21	[21]	Not primary focus	Genetic markers associated with trans FA content in milk; Identification of 68 SNPs linked to trans FA across breeds	Not primary focus	GWAS, SNP mapping
22	[22]	Diet and breed affect bovine milk composition	Breed differences in low abundance of milk particular components identified	Lactation stage and diet modulate particular components profile	LC-MS
23	[23]	Multi- factors influencing EFA and CLA effects on dairy cow physiology	Not primary focus	Transition period physiological changes emphasized	Molecular phenotyping review
24	[24]	Palmitic to oleic acid ratio effects on early lactation cows; Not gene-focused; Increasing oleic acid improves production and milk FA profile	Not primary focus	Early lactation physiological responses measured	Nutrient digestibility, FA profiling
25	[25]	Lipid source effects on intake and milk yield in crossbred cows. Soybean oil increases digestibility; cottonseed reduces milk yield	Not gene-focused; crossbred cows studied	Early lactation	*In vitro* digestibility, PCA

No.	Reference	Dietary Influence	Genetic Variation	Environmental and Physiological Effects	Analytical Methodologies
26	[26]	Whole *vs* ground flaxseed effects on milk n-3 PUFA; Ground flaxseed is more effective in increasing milk n-3 PUFA	Not gene-focused; breed Holstein	Lactation stage mid	LC-MS/MS,plasma and milkFA
27	[27]	Not primary focus	Milk composition differences linked to species; Buffalo vs cow milk differentially affects lipid metabolism in obese mice	Not primary focus	LC-MS/MS, gene expression
28	[28]	Not primary focus	Breed differences significant but limited; local *vs* cosmopolitan breeds of goat	Lactation week affects FA profile more than breed	GC-MS, mixed model analysis
29	[29]	Not primary focus	GWAS of genes affecting de novo milk FA synthesis; Candidate genes PAEP, ACSS2,NCOA6, FASN identified	Not primary focus	GWAS, RNA-seq
30	[30]	Not primary focus	Not gene-focused; Species and breed not varied; focus on maternal-neonate lipid transfer; milk and plasma lipidome comparison in lambs of sheep	Lactation based, Lactation day post-birth studied in terms of milk profile and impact	Untargeted mass spectrometry
32	[32]	Not primary focus	Not primary focus	Season affects milk fat crystallization properties and bovine milk TAG profile	MALDI-TOF/TOF MS

Table S1. Intrinsic and Extrinsic Factors impact on Milk Lipid Profiles

No.	Reference	Dietary Intervention Type	Milk Fatty Acid Composition	Rumen Biohydrogenation Modulation	Animal Factors Influence	Health/Technology Related Lipid Outcomes
1	[17]	Lipid supplementation with varied FA profiles	SFA diets increase milk fat; UFA diets increase long-chain FA	Biohydrogenation affects FA transfer	Diet and animal production traits influence	Predictive models for milk FA profile
2	[18]	Agro-industrial by-products (grape, pomegranate, olive, tomato)	Positive effects on milk FA profile without depressing yield	Polyphenols modulate rumen biohydrogenation	Multi-species difference (cow, sheep, goat)	Enhanced nutritional quality and sustainability
3	[33]	Forage-based diets and nutrition modeling	Grass milk has lower omega-6/omega-3 ratio	Forage type influences rumen biohydrogenation	Not primary focus	Potential cardiovascular benefits
4	[34]	SFA-reduced dairy consumption	Reduced LDL cholesterol with modified dairy	Not primary focus	Not primary focus	Cardiovascular risk marker improvement
5	[35]	Dietary modifications and genetic manipulation	Altered SFA and PUFA proportions	Rumen microbiota impacts FA profile	Genetic selection potential	Health benefits linked to FA profile
6	[36]	Vegetable seeds, oils, microalgae, phytogenics	Increased n-3 FA,CLA, unsaturated FA	Feeding strategies alter biohydrogenation	Not primary focus	Enrichment of anticarcinogenic FA
7	[37]	Oilseed supplementation (linseed, soybean, rapeseed)	Increased CLA,VA, OA, LNA, UFA; decreased SFA	Processing and type affect biohydrogenation	Breed, lactation stage affect response	Improved milk components for health
8	[38]	Oilseeds, vegetable oils, bypass fats	Increased MUFA and PUFA; decreased SFA	Rumen biohydrogenation limits unsaturated FA	Not primary focus	Opportunities for nutraceutical milk
9	[39]	Protected palm fats in crossbred cows	Decreased SFA, increased MUFA and PUFA	Not primary focus	Breed and lactation stage considered	Enhanced cardio-protective milk fat
10	[40]	Sunflower oil with selenium and vitamin E	Increased CLA, vaccenic acid; reduced milk fat content	Antioxidants may influence rumen environment	Lactation stage considered	Anticarcinogenic and antioxidan benefits
11	[41]	trans-10, cis-12 CLA effects	Dose-dependent milk fat synthesis inhibition	Not primary focus	Not primary focus	Minimal CLA inhibits milk fat

No.	Reference	Dietary Intervention Type	Milk Fatty Acid Composition	Rumen Biohydrogenation Modulation	Animal Factors Influence	Health/Technology Related Lipid Outcomes
12	[42]	Fat sources, technological forms, basal diet	Fat form and forage type affect milk FA profile	Protected fats alter biohydrogenation	Forage type modulates effects	Optimized milk fat quality
13	[43]	Linseed treatments and DHA addition	Reduced biohydrogenation of C18:3n3 with treatments	pH and fat form affect biohydrogenation	Forage type and concentrate ratio influence	Enhanced unsaturated FA secretion
14	[44]	Milk FA and brain metabolism	Feeding systems impact milk FA quality	Rumen biohydrogenation modulated by diet	Species and rearing system effects	Potential CNS benefits
15	[45]	Echium oil supplementation and genetics	Increased n-3 FA and CLA via diet and genetics	Rumen microbial shifts affect FA profile	Breed and lactation stage significant	Enhanced bioactive FA in milk
16	[46]	Milk FA profile and energy balance	NEB affects milk FA composition dynamically	Not primary focus	Lactation stage critical	Milk FA as energy status indicator
17	[47]	Marine lipids, plant compounds, microbials	Increased desirable FA like CLA, vaccenic acid	Modulation of rumen lipid metabolism	Species and diet dependent	Challenges inside effects and stability
18	[48]	CLA biosynthesis in ruminants and humans	Endogenous synthesis major CLA source	Rumen biohydrogenation complex and variable	Not primary focus	Dietary prediction complicated
19	[49]	Improved milk FA profile with camelina, linseed, sunflower	Oilseed industry byproducts rich in linoleic and linolenic acids	Limited direct rumen modulation data	Species differences noted (cow, sheep, goat)	Potential for healthier milk fat
20	[50]	Alternative feeds: tanniferous plants, herbs, algae	Increased PUFA, n-3 FA, CLA in milk fat	Secondary compounds affect rumen microbes	Not primary focus	Improved health properties of milk fat
21	[51]	EFA and CLA supplementation during transition period	EFA and CLA supplementation during transition period	Biohydrogenation variability affects outcomes	Transition stage critical	Improved energy balance and immunity, Modulation of immune and energy metabolism
22	[52]	Pasture and oil supplementation; genetic approaches	Enhanced CLA, omega-3, reduced SFA	Rumen biohydrogenation pathways detailed	Genetic selection potential	Improved milk nutritional quality

No.	Reference	Dietary Intervention Type	Milk Fatty Acid Composition	Rumen Biohydrogenation Modulation	Animal Factors Influence	Health/Technology Related Lipid Outcomes
23	[53]	Forage-to-concentrate ratio, pasture, dietary fat	Increased CLA, omega-3; decreased SFA with pasture	Forage type influences biohydrogenation	Genetic improvement demonstrated	Nutritional quality enhancement
24	[54]	Oilseed type and processing in commercial farms	Decreased SFA, increased MUFA and PUFA	Farm management affects biohydrogenation	Breed and lactation stage considered	Practical application in commercial settings
25	[55]	PUFA-rich lipid sources and bacterial strains	Increased CLA content in milk and dairy products	Biohydrogenation and fermentation processes	Genetic factors also contribute	Effective CLA enrichment strategies
26	[56]	Dietary manipulation and processing technologies; Dietary lipids modulate milk fat composition	Modified milk fat for health and functionality	Not primary focus	Not primary focus	Health benefits and product innovation
27	[57]	Palmitic acid and n-6:n-3 ratio alteration	Increased milk yield; improved FA profile	Not primary focus	Low-fiber diets and lactation stage	Enhanced feed efficiency and milk quality
28	[58]	Feeding strategies in sheep for milk FA	Increased CLA and omega-3 in milk and cheese	Limited rumen biohydrogenation data	Species-specific responses	Cost-effective nutritional improvements
29	[59]	Oilseed supplementation to reduce SFA	Reduced SFA and trans FA with protected oilseeds	Protection from rumen metabolism critical	Not primary focus	Improved nutritive quality of milk
30	[60]	Different lipid sources in lactating cow diets	Varied effects on milk yield, CLA, and FA profile	Lipid source influences biohydrogenation	Small sample size; breed considered	Milk composition modulation
31	[61]	Grazing combined with total mixed ration	Increased CLA, vaccenic acid, and omega-3	Fresh forage enhances biohydrogenation intermediates	Grazing time and lactation stage	Healthier milk fat profile
32	[62]	Nutrition effects on cow and goat milk FA; Oilseed supplements affect trans isomers	Grass-based diets reduce SFA, increase CLA	Not primary focus	Species differences in response	Milk authentication and methane prediction

No.	Reference	Dietary Intervention Type	Milk Fatty Acid Composition	Rumen Biohydrogenation Modulation	Animal Factors Influence	Health/Technology Related Lipid Outcomes
33	[63]	High concentrate and fat supplementation; Complementary effects on trans FA	Modified milk FA profile without yield loss	Not primary focus	Not primary focus	Nutritional value enhancement
34	[64]	Lipid supplementation in goats; Protected fats improve FA profile	Increased PUFA and CLA in milk	Not primary focus	Goat-specific responses	Functional food potential
35	[65]	FA variability sources and impacts	Genetics, nutrition, season affect milk FA	Farming system influences biohydrogenation	Breed and lactation stage important	Technological and health applications
36	[66]	Milk fat composition and functionality	Reduced SFA and increased omega-3 and CLA	Not primary focus	On-farm and processing strategies	Nutritional and physical improvements
37	[67]	Feeding oilseeds to alter milk FA	Protection from rumen biohydrogenation critical	Not primary focus	Not primary focus	Improved nutritive quality
38	[68]	Sheep and goat feeding strategies	Sheep respond better to PUFA-rich diets	Polyphenols modulate rumen biohydrogenation	Species differences in response	Improved oxidative stability
39	[69]	DHA-rich microalgae supplementation; Pelleting affects DHA transfer	Increased DHA and lowered n-6:n-3 ratio	Not primary focus	Not primary focus	Suitable for cheese production
40	[70]	Dietary FA and cardiometabolic health	Replacement of SFA with unsaturated FA	Rumen biohydrogenation affects TFA	Not primary focus	Cardiometabolic health benefits
41	[71]	Linoleic and linolenic acid effects	Increased CLA productionmainly from linoleic acid	Endogenous synthesis major CLA source	Individual cow variation	Enhanced milk CLA content
42	[72]	Feeding to enhance milk and meat FA	Grass and supplements increase PUFA and CLA	Biohydrogenation intermediates vary by diet	Species differences in metabolism	Lipid peroxidation risk managed by antioxidants
43	[73]	Feeding effects on sheep milk and cheese	Grazing and oil supplements increase CLA andomega-3	Limited rumen biohydrogenation data	Species-specific responses	Nutritional value improvement

No.	Reference	Dietary Intervention Type	Milk Fatty Acid Composition	Rumen Biohydrogenation Modulation	Animal Factors Influence	Health/Technology Related Lipid Outcomes
44	[74]	Enhancing milk FA beneficial to health	Dietary lipids increase CLA, EPA, DHA	Rumen biohydrogenation limits transfer	Individual cow differences	Significant enhancement possible
45	[75]	Factors affecting milk liposoluble components; Forage and lipid supplements modify FA	Breed, lactation, and diet influence milk FA	Not primary focus	Species and breed differences	Improved consumer health potential
46	[76]	Genetic and dietary effects on milk FA	Genetic variation affects milk FA profile	Not primary focus	Breed and lactation stage important	Healthier milk fat achievable
47	[77]	Animal multi-factors on milk FA composition	Breed, parity, lactation stage affect FA profile	Not primary focus	Genetic polymorphisms, Breed, parity, lactation stage influence FA variability; Individual cow differences significant	Breeding for improved milk FA
48	[78]	Lipid supplementation effects	Protected fats increase long-chain FA	Not primary focus	Not primary focus	Milk fat composition modulation
49	[79]	Palm kernel cake supplementation	Altered milk and cheese FA profile negatively	Not primary focus	Not primary focus	Increased lauric and tridecanoic acids
50	[80]	Milk fat research targets	Molecular mechanisms complex	Milk fat depression linked to biohydrogenation	Not primary focus	Multivariate approaches needed
51	[81]	Nutritional control of milk FA	Diet affects milk CLA and PUFA content	Forage and fat supplements modulate biohydrogenation	Not primary focus	Potential 300% CLA increase
52	[82]	Abomasal infusion of seed and fish oils	Olive oil increases oleic acid in milk	Intestinal digestibility affects milk FA	Not primary focus	Olive oil best for health benefits
53	[83]	Dairy fat perceptions and realities	Saturated and trans fats in milk debated	Dietary formulations bypass rumen biohydrogenation	Not primary focus	Dairy foods critical nutrient sources
54	[84]	CLA infusion effects on milk	Increased milk CLA but reduced milk fat	Not primary focus	Not primary focus	Specific CLA isomers inhibit milk fat
55	[85]	Milk lipid composition and absorption	Milk fat contains bioactive lipids with health effects	Diet and rumen influence milk fat composition	Species differences important	Personalized nutrition implications

No.	Reference	Dietary Intervention Type	Milk Fatty Acid Composition	Rumen Biohydrogenation Modulation	Animal Factors Influence	Health/Technology Related Lipid Outcomes
56	[86]	Dairy phospholipids in human nutrition	MFGM phospholipids have nutraceutical properties	Limited rumen focus	Not primary focus	Cardiovascular and cognitive benefits
57	[87]	Grass and maize diets alter milk FA; Milk rich in protein, low fat; Oil supplementation effects variable	Grass and maize diets alter milk FA	Not primary focus	Not primary focus	Increased unsaturated FA proportions
58	[88]	Fish oil supplementation effects; Protected fish oil reduces negative effects	Fish oil increases n-3 FA but reduces milk fat	Not primary focus	Not primary focus	Milk oxidation and taste affected
59	[89]	Partial SFA replacement in dairy	MUFA-rich diets reduce LDL cholesterol	Limited rumen focus	Not primary focus	Beneficial cardiovascular effects
60	[90]	DHA transfer efficiency	Transfer efficiency declines with higher DHA intake	Not primary focus	Not primary focus	DHA transfer to milk limited
61	[91]	Mathematical model of FA synthesis;Rate-limiting steps identified	SCD upregulation modestly increases MUFA	Not primary focus	Genetic mutation effects modeled	Potential for low-fat milk production
62	[92]	Milk lipid secretion regulation; Dietary trans fats may depress milk lipid	FA regulate mammary lipogenesis	Not primary focus	Not primary focus	Hormonal and enzymatic control
63	[93]	Protected lipid feeding effects; Lipoprotein changes affect milk FA	Increased long-chain FA in milk and serum	Not primary focus	Not primary focus	Milk fat composition altered
64	[94]	Genetic and dietary factors on milk FA	DGAT-1 and SCD genes affect FA profile	Not primary focus	Breed and parity effects; Genetic variation influences milk FA	Healthier milk fat composition
65	[95]	Pasture feeding, lipid supplements, breed effects	Enhanced CLA and omega-3 with pasture	Seasonal and regional variations affect biohydrogenation	Breed, lactation stage influence	Milk differentiation for health
66	[96]	Nutritional strategies for designer milk	Feeding increases MUFA and PUFA in milk	Rumen metabolism affects FA profile	Not primary focus	Healthier milk for consumers
67	[97]	CLA supplementation effects	CLA increases milk CLA but reduces milk fat	Abomasal infusion bypasses rumen	Not primary focus	Milk fat synthesis inhibition
68	[98]	Increasing DHA and EPA in milk	Marine oils and microalgae increase n-3 FA	Rumen biohydrogenation limits transfer	Not primary focus	Protection and transport challenges

Table S2. Intrinsic and Extrinsic Factors impact including Rumen Biohydrogenation on Fatty Acids compositon

Area of Limitation	Description of Limitation	Papers which have limitation
**Heterogeneity in study designs**	Many studies exhibit high heterogeneity in experimental designs, including variations in animal breeds, lactation stages, oilseed types used for feed fortification, and processing methods of the supplement. This variability limits the external validity and comparability of findings across studies.	[17,37,43]
**Small or uneven sample sizes**	Several investigations utilize relatively small sample sizes or uneven group distributions, which may reduce statistical power and increase the risk of type II errors, thereby weakening the robustness of conclusions regarding milk lipid profiles.	[6,7,22,28,45,77]
**Limited control groups**	Several meta-analyses and reviews exclude studies lacking proper control groups, which restricts the comprehensiveness of the evidence base and may introduce selection bias, affecting the robustness of conclusions.	[37]
**Incomplete reporting of variables**	Unknown or unreported factors such as diet composition, animal characteristics, and experimental conditions contribute to unexplained variability, weakening the ability to generalize results and identify causal relationships.	[37,45]
**Short duration of interventions**	Many feeding trials and supplementation studies have relatively short durations, limiting the understanding of long-term effects on milk fatty acid profiles and animal health, thus constraining the applicability of findings to commercial settings.	[45,69]
**Limited breed and species diversity**	Research on milk lipid profiles is predominantly focused on a narrow range of breeds or crossbreeds, particularly the Holstein breed, leading to a significant limitation in the external validity of findings (e.g., lipid and proteome variations in milk). This constrained focus limits the generalizability of results to diverse dairy populations and management systems, as there is limited data on other breeds or species (e.g., specific genetic backgrounds) that might exhibit different responses to dietary or environmental manipulation.	[1,6,7,19,28,37,45,99]
**Variability in dietary lipid sources**	Differences in lipid sources, their processing, and inclusion levels across studies introduce variability that complicates the synthesis of results and practical recommendations for milk lipid profile manipulation.	[37,42,60]
**Lack of standardization in dietary interventions**	Variability in diet composition, supplementation levels, and feeding regimes across studies introduces confounding factors, reducing comparability and complicating the synthesis of dietary effects on milk lipid profiles.	[1,15–17]
**Challenges in rumen protection**	Effective rumen protection of unsaturated fatty acids remains difficult, with many technologies failing to prevent biohydrogenation fully, thereby limiting the transfer efficiency of beneficial fatty acids into milk and affecting study outcomes.	[45]
**Genetic marker limitations**	Genetic association studies often focus on a limited set of candidate genes or SNPs, with an incomplete understanding of causal variants, which constrains the ability to fully exploit genetic selection for milk lipid profile manipulation.	[20,21,29,35]
**Potential negative effects on milk quality**	Some dietary interventions, such as fish oil supplementation, may adversely affect milk taste, oxidative stability, and yield, posing challenges for commercial adoption and consumer acceptance.	[88]
**Limited longitudinal data**	Many studies provide cross-sectional or short-term data, limiting understanding of dynamic changes in milk lipid and protein profiles across full lactation cycles or multiple seasons, which affects temporal validity.	[1,6,14,28]

Area of Limitation	Description of Limitation	Papers which have limitations
**Methodological variability**	Differences in analytical techniques (e.g., GC-FID,HPLC-MS, MALDI-TOF, MIRS) and calibration approaches introduce methodological constraints, complicating direct comparisons and potentially affecting the accuracy of lipid and protein quantifications.	[1][7][35][22]
**Geographic and environmental bias**	Most studies are conducted in specific regions or controlled environments, which may not capture the full range of environmental influences on milk lipid composition, thus limiting ecological validity.	[1,12,19,28]
**Insufficient focus on microbial modulation**	Research on how milk lipids modulate bacterial protein expression remains sparse, limiting insights into functional interactions critical for dairy applications and microbial ecology.	[10,11]

Table S3. Limitations of the Literature

Gap Area	Description	Future Research Directions	Justification	Research Priority
**Mechanistic links & multi-omics integration**	Limited direct evidence on how specific milk lipid profiles modulate bacterial protein expression in dairy applications. Current studies also rarely integrate multi-omics data (lipidomics, genomics, proteomics, microbial omics) to comprehensively understand these interactions.	Conduct integrative *in vitro* and *in vivo* studies combining milk lipidomics, host transcriptomics, and microbial proteomics/metagenomics to elucidate causal mechanisms and unravel complex, multifactorial interactions influencing dairy product quality.	Multi-omics integration is necessary to capture the multifactorial nature of milk lipid effects on microbial functions, which is critical for leveraging milk lipids to control microbial activity and improve dairy product quality [10,11,13,23].	High
**Genetic/nutritional interactions & breed variation**	Genetic factors (e.g., SCD, DGAT-1 polymorphisms) are often studied in isolation, and gene-diet interactions remain incompletely elucidated. Studies often focus on a limited number of breeds, lacking functional validation and limiting the generalizability of findings.	Expand genomic and transcriptomic analyses to diverse breeds (including indigenous and crossbreeds). Develop combined breeding and feeding programs that consider gene-diet interactions, performing GWAS linked with dietary trials to optimize milk fat quality and validate candidate gene functions using gene editing.	Understanding gene-diet interplay and breed-specific genetic architecture is crucial for precision nutrition and breeding to enhance milk quality, optimize microbial interactions, and improve breeding strategies [4,20,21,52,77,94].	High
**Incomplete Rumen Biohydrogenation Understanding**	The complexity and variability of rumen microbial biohydrogenation limit consistent enhancement of unsaturated fatty acids (UFA) in milk. Additionally, the contribution of rumen protozoa to biohydrogenation intermediates and UFA protection is underexplored.	Conduct longitudinal and mechanistic studies to characterize rumen microbial populations, their biohydrogenation pathways, and protozoal species-specific roles under diverse diets. Develop targeted microbial or dietary interventions to optimize biohydrogenation.	Variability in rumen microbial responses and the underexplored role of protozoa hinders the practical application of dietary strategies and limits consistent milk FA profiles [43,45,47].	High
**Limited efficacy of rumen-protection technologies**	Current rumen-protection methods inadequately prevent biohydrogenation and often reduce intestinal digestibility of PUFA, resulting in low transfer efficiency to milk fat.	Innovate and test novel rumen-protection technologies that balance protection from biohydrogenation with efficient intestinal release and absorption; evaluate long-term effects on milk fatty acid composition.	Low transfer efficiency of PUFA into milk fat due to incomplete rumen protection and poor intestinal availability restricts milk enrichment [45].	High
**Impact of dietary lipids on microbial modulation**	While dietary effects on milk lipid profiles are documented, their downstream impact on microbial protein expression during dairy	Investigate how specific dietary lipid sources alter milk lipid composition and subsequently affect microbial gene and protein expression during	Linking diet to microbial functional outcomes via milk lipids can optimize feeding strategies for improved dairy	High

Gap Area	Description	Future Research Directions	Justification	Research Priority
	fermentation and gut colonization is poorly understood.	dairy fermentation and gut colonization.	product functionality [5,15,16].	
**Clinical and commercial feasibility**	Insufficient human clinical evidence links modified milk fat to health outcomes. Furthermore, some interventions cause milk fat depression and sensory changes, limiting commercial feasibility.	Design and implement randomized controlled trials (RCTs) to assess cardiometabolic and inflammatory outcomes in humans consuming modified milk. Explore dose-response relationships to minimize milk fat depression and develop processing methods to maintain sensory quality.	The translational gap limits validation of health benefits. Milk fat depression reduces yield and alters quality, limiting commercial feasibility [40,70,88,100–102].	High
**Standardization of analytical methodologies**	Variability in sample preparation, lipid extraction, and analytical platforms hampers comparability across studies, especially for linking lipidomics to functional assays.	Develop and validate standardized protocols for milk lipid extraction and multi-omics analyses, including integration with bacterial protein expression assays, to enable reproducible and comparable results.	Standardization is essential to build a coherent knowledge base and facilitate meta-analyses and cross-study comparisons [1,103].	High
**Longitudinal and Physiological Studies**	Most studies assess lactation stage effects cross-sectionally, limiting understanding of individual animal lipid profile trajectories. Effects of parity, individual variation, and response to diet are also not fully elucidated.	Design longitudinal cohort studies tracking individual animals across lactation stages and physiological states with repeated milk lipid and proteome profiling, coupled with microbial functional assays.	Longitudinal data will clarify temporal dynamics of milk lipids, energy balance, and their functional roles, improving strategies for milk quality optimization and optimizing feeding strategies (Kostovska et al., 2024; Sinanoglou et al., 2015; Rodríguez-Bermúdez et al., 2023; Gross et al., 2011; Bainbridge, 2017; Samková et al., 2012).	Medium
**Influence of environmental and welfare factors**	Environmental and welfare factors are underrepresented and insufficiently quantified, limiting understanding of their effects on milk lipid profiles and microbial modulation.	Implement controlled experiments and field studies quantifying environmental variables (e.g., temperature, pasture composition) and welfare indicators, assessing their direct and interactive effects on milk lipids and bacterial protein expression.	These factors contribute to milk composition variability and may indirectly affect microbial interactions; better quantification will improve management practices [1,2].	Medium
**Functional validation of milk lipid fractions**	Few studies assess the biological effects of specific milk lipid fractions on host intestinal cells and dairy-relevant microbes.	Perform targeted functional assays using isolated milk lipid fractions on relevant bacterial strains and intestinal cell models to evaluate effects on protein expression, viability, and immune modulation.	Functional validation will clarify the health and technological implications of milk lipids beyond compositional analysis [5,104].	Medium

Gap Area	Description	Future Research Directions	Justification	Research Priority
**Cross-species and breed comparative analyses**	Comparative studies across species and breeds are limited by inconsistent methodologies and lack of focus on microbial modulation by milk lipids.	Conduct standardized comparative studies across multiple species and breeds using harmonized lipidomic and microbial functional assays to identify species- and breed-specific lipid-microbe interactions.	Comparative insights can guide breed selection and dairy product development tailored to functional lipid profiles [19,28,105]..	Medium
**Underutilization of agro-industrial by-products**	The potential of agro-industrial by-products and unconventional feeds to improve milk fatty acid profiles is promising but under-researched for optimal inclusion and effects.	Systematically evaluate different by-products and alternative feeds for their effects on rumen biohydrogenation, milk fatty acid profiles, and animal performance across species.	By-products offer sustainable options to enhance milk fat quality without depressing production, but require further validation [18,50].	Medium
**Limited mechanistic modeling**	Existing models of mammary fatty acid synthesis and enzyme regulation are insufficiently detailed to predict complex dietary and genetic interactions.	Develop advanced mechanistic and systems biology models incorporating rumen metabolism, mammary gland enzymatic activity, and genetic factors to predict milk fatty acid outcomes.	Improved models can guide targeted interventions and breeding for desired milk fat composition [17,c91].	Medium
**Insufficient understanding of MFGM phospholipids role**	The role of Milk Fat Globule Membrane (MFGM) phospholipids in human nutrition and their modulation through diet is poorly understood.	Conduct *in vivo* human studies and animal trials to elucidate the health effects of dairy phospholipids and explore dietary strategies to enhance their content in milk.	MFGM phospholipids have nutraceutical potential but are understudied compared to fatty acid profiles [86].	Low

Table S4. Gaps and Future Research Directions

Reference

1. Kostovska, R.; Drouin, G.; Salas, J.J.; Venegas-Calerón, M.; Horan, B.; Tobin, J.T.; O'Callaghan, T.F.; Hogan, S.A.; Kelly, A.L.; Gómez-Mascaraque, L.G. Multispecies pasture diet and cow breed affect the functional lipid profile of milk across lactation in a spring-calving dairy system. *J. Dairy Sci.* **2025**, *108*, 1261–1284, doi:10.3168/jds.2024-25462.
2. Cozma, A.; Miere, D.; Filip, L.; Banc, R.; Stanciu, O.; Andrei, S.; Loghin, F. Factors Influencing the Concentration of Certain Liposoluble Components in Cow and Goat Milk: A Review. *Not. Sci. Biol.* **2014**, *6*, 267–272, doi:10.15835/nsb639404.
3. Kala, R.; Samková, E.; Koubová, J.; Hasoňová, L.; Kvác, M.; Pelikánová, T.; Spicka, J.; Hanus, O. Nutritionally desirable fatty acids including CLA of cow⇔s milk fat explained by animal and feed factors. *Acta Univ. Agric. Silvic. Mendelianae Brun.* **2018**, *66*, 69–76, doi:10.11118/actaun201866010069.
4. Maurice – Van Eijndhoven, M. *Genetic variation of milk fatty acid composition between and within dairy cattle breeds*; 2014; ISBN 9789462571488.
5. Sun, X.; Wang, Y.; Ma, X.; Li, S.; Wang, W. Producing natural functional and low-carbon milk by regulating the diet of the cattle—The fatty acid associated rumen fermentation, biohydrogenation, and microorganism response. *Front. Nutr.* **2022**, *9*, 1–18, doi:10.3389/fnut.2022.955846.
6. Sinanoglou, V.J.; Koutsouli, P.; Fotakis, C.; Sotiropoulou, G.; Cavouras, D.; Bizelis, I. Assessment of lactation stage and breed effect on sheep milk fatty acid profile and lipid quality indices. *Dairy Sci. Technol.* **2015**, *95*, 509–531, doi:10.1007/s13594-015-0234-5.
7. Devi, S.;Kapila, R. Kapila, S. Interbreed Variation of Dairy Cow Milk in Terms of Specific Proteome and Lipidome for Establishing Criteria of Milk Selection. *Nutr. Health* **2025**, doi:doi:10.1177/02601060241307063.
8. van Arendonk, J.A.M.; van Valenberg, H.J.F.; Bovenhuis, H. *Exploiting genetic variation in milk-fat composition of milk from dairy cows*; Woodhead Publishing Limited, 2010; Vol. 1; ISBN 9781845694388.
9. Verma, A.; Meitei, N.S.; Gajbhiye, P.U.; Raftery, M.J.; Ambatipudi, K. Comparative analysis of milk triglycerides profile between jaffarabadi buffalo and holstein friesian cow. *Metabolites* **2020**, *10*, 1–27, doi:10.3390/metabo10120507.
10. Siurana, A.; Cánovas, A.; Casellas, J.; Calsamiglia, S. Transcriptome Profile in Dairy Cows Resistant or Sensitive to Milk Fat Depression. *Animals* **2023**, *13*, 1–11, doi:10.3390/ani13071199.
11. Suárez-Vega, A.; Gutiérrez-Gil, B.; Toral, P.G.; Frutos, P.; Loor, J.J.; Arranz, J.J.; Hervás, G. Elucidating genes and gene networks linked to individual susceptibility to milk fat depression in dairy goats. *Front. Vet. Sci.* **2022**, *9*, doi:10.3389/fvets.2022.1037764.
12. Rodríguez-Bermúdez, R.; Fouz, R.; Rico, M.; Camino, F.; Souza, T.K.; Miranda, M.; Diéguez, F.J. Factors Affecting Fatty Acid Composition of Holstein Cow's Milk. *Animals* **2023**, *13*, 1–12, doi:10.3390/ani13040574.
13. Giannuzzi, D.; Toscano, A.; Pegolo, S.; Gallo, L.; Tagliapietra, F.; Mele, M.; Minuti, A.; Trevisi, E.; Marsan, P.A.; Schiavon, S.; et al. Associations between Milk Fatty Acid Profile and Body Condition Score, Ultrasound Hepatic Measurements and Blood Metabolites in Holstein Cows. *Animals* **2022**, *12*, doi:10.3390/ani12091202.
14. Veshkini, A.; Hammon, H.M.; Vogel, L.; Viala, D.; Delosière, M.; Tröscher, A.; Déjean, S.; Ceciliani, F.; Sauerwein, H.; Bonnet, M. The skimmed milk proteome of dairy cows is affected by the stage of lactation and by supplementation with polyunsaturated fatty acids. *Sci. Rep.* **2024**, *14*, 1–13, doi:10.1038/s41598-024-74978-1.
15. Wang, Jiaqi, Liu, Kaizhen and Chen, Meiqing and Huang, Guoxin and Su, Chuanyou and Tang, Wenhao and Li, Ning and Wu, Xufang and Si, Boxue and Zhao, Shengguo and Zheng, Nan and Zhang, Yangdong and Wang, Jiaqi Variations of Milk Lipidomic Profile in Dairy Cow Fed Alfalfa Hay or Alfalfa Silage as Roughage. *KeAi* **2023**, 1–35, doi:10.2139/ssrn.4612883.

16. Bhavsar, M.Y.; Pandya, P.R.; Patel, Y.G.; Shah, S. V.; Bhavsar, P.P. Effects of Feeding Flaxseeds and Rapeseeds on Milk Fatty Acid Composition in Crossbred Dairy Cattle. *Indian J. Vet. Sci. Biotechnol.* **2023**, *19*, 51–56, doi:10.48165/ijvsbt.19.1.12.
17. Gallardo, W.B.; Teixeira, I.A.M.A. Associations between Dietary Fatty Acid Profile and Milk Fat Production and Fatty Acid Composition in Dairy Cows: A Meta-Analysis. *Animals* **2023**, *13*, doi:10.3390/ani13132063.
18. Correddu, F.; Caratzu, M.F.; Nudda, A.; Lunesu, M.F.; Carta, S.; Pulina, G. Grape, Pomegranate, Olive, and Tomato By-Products Fed to Dairy Ruminants Improve Milk Fatty Acid Profile without Depressing Milk Production. *Foods* **2023**, doi:doi.org/10.3390/foods12040865.
19. Conte, G.; Palombo, V.; Serra, A.; Correddu, F.; D'andrea, M.; Macciotta, N.P.P.; Mele, M. Study of the Fatty Acid Profile of Milk in Different Sheep Breeds: Evaluation by Multivariate Factorial Analysis. *Animals* **2022**, *12*, 1–14, doi:10.3390/ani12060722.
20. Carvajal, A.M.; Huircan, P.; Dezamour, J.M.; Subiabre, I.; Kerr, B.; Morales, R.; Ungerfeld, E.M. Milk fatty acid profile is modulated by DGAT1 and SCD1 genotypes in dairy cattle on pasture and strategic supplementation. *Genet. Mol. Res.* **2016**, *15*, doi:10.4238/gmr.15027057.
21. Bykova, O.; Shevkunov, O.; Kostyunina, O. Overview of SNPs Associated with Trans Fat Content in Cow's Milk. *Agric.* **2023**, *13*, 1–18, doi:10.3390/agriculture13061151.
22. Tacoma, R. Examination of the effects breed and nutrition have on the milk protein profile produced by lactating dairy cattle. **2016**.
23. Veshkini, A.; Ceciliani, F.; Bonnet, M.; Hammon, H.M. Review: Effect of essential fatty acids and conjugated linoleic acid on the adaptive physiology of dairy cows during the transition period. *Animal* **2023**, *17*, 100757, doi:10.1016/j.animal.2023.100757.
24. Hu, L.; Shen, Y.; Zhang, H.; Ma, N.; Li, Y.; Xu, H.; Wang, M.; Chen, P.; Guo, G.; Cao, Y.; et al. Effects of dietary palmitic acid and oleic acid ratio on milk production, nutrient digestibility, blood metabolites, and milk fatty acid profile of lactating dairy cows. *J. Dairy Sci.* **2024**, *107*, 4370–4380, doi:10.3168/jds.2023-23801.
25. Wanderley, A.M.; Ítavo, L.C.V.; dos Santos, G.T.; Ítavo, C.C.B.F.; Cunha, C.S.; dos Santos Difante, G.; Dias, A.M.; Mateus, R.G.; de Oliveira, M.V.M. Ruminal degradation kinetics of diets with different lipid sources and its influence on intake and milk yield of early lactation crossbred Holstein × Gir cows. *Trop. Anim. Health Prod.* **2021**, *53*, doi:10.1007/s11250-021-02960-4.
26. Huang, G.; Wang, J.; Liu, K.; Wang, F.; Zheng, N.; Zhao, S.; Qu, X.; Yu, J.; Zhang, Y.; Wang, J. Effect of Flaxseed Supplementation on Milk and Plasma Fatty Acid Composition and Plasma Parameters of Holstein Dairy Cows. *Animals* **2022**, *12*, doi:10.3390/ani12151898.
27. Jiang, M.; Meng, Z.; Cheng, Z.; Zhan, K.; Ma, X.; Yang, T.; Huang, Y.; Yan, Q.; Gong, X.; Zhao, G. Effects of Buffalo Milk and Cow Milk on Lipid Metabolism in Obese Mice Induced by High Fat. *Front. Nutr.* **2022**, *9*, 1–10, doi:10.3389/fnut.2022.841800.
28. Currò, S.; Manuelian, C.L.; De Marchi, M.; Claps, S.; Rufrano, D.; Neglia, G. Effects of breed and stage of lactation on milk fatty acid composition of Italian goat breeds. *Animals* **2019**, *9*, 1–16, doi:10.3390/ani9100764.
29. Knutsen, T.M.; Olsen, H.G.; Tafintseva, V.; Svendsen, M.; Kohler, A.; Kent, M.P.; Lien, S. Unravelling genetic variation underlying de novo-synthesis of bovine milk fatty acids. *Sci. Rep.* **2018**, *8*, 1–13, doi:10.1038/s41598-018-20476-0.
30. Thangaraj, S.V.; Ghnenis, A.; Pallas, B.; Vyas, A.K.; Gregg, B.; Padmanabhan, V. Comparative lipidome study of maternal plasma, milk, and lamb plasma in sheep. *Sci. Rep.* **2024**, *14*, 1–15, doi:10.1038/s41598-024-58116-5.
32. Tzompa-Sosa, D.A.; Meurs, P.P.; van Valenberg, H.J.F. Triacylglycerol Profile of Summer and Winter Bovine Milk Fat and the Feasibility of Triacylglycerol Fragmentation. *Eur. J. Lipid Sci. Technol.* **2018**, *120*, 1–13, doi:10.1002/ejlt.201700291.

33. Benbrook, C.M.; Davis, D.R.; Heins, B.J.; Latif, M.A.; Leifert, C.; Peterman, L.; Butler, G.; Faergeman, O.; Abel-Caines, S.; Baranski, M. Enhancing the fatty acid profile of milk through forage-based rations, with nutrition modeling of diet outcomes. *Food Sci. Nutr.* **2018**, *6*, 681–700, doi:10.1002/fsn3.610.
34. Vasilopoulou, D.; Markey, O.; Fagan, C.C.; Kliem, K.E.; Humphries, D.J.; Jackson, K.G.; Todd, S.; Givens, D.I.; Lovegrove, J.A. Chronic consumption of conventional and saturated-fat reduced dairy products have differential effects on low-density lipoprotein cholesterol levels in adults at moderate cardiovascular disease risk. *Proc. Nutr. Soc.* **2016**, *75*, 2089035, doi:10.1017/s0029665116001841.
35. Santin Junior, I.; Silva, K.; Cucco, D. Milk Fatty Acids Profile and the Impact on Human Health. *J. Dairy Vet. Sci.* **2019**, *10*, doi:10.19080/jdvs.2019.10.555779.
36. Kholif, A.E.; Olafadehan, O.A. Dietary strategies to enrich milk with healthy fatty acids - A review. *Ann. Anim. Sci.* **2022**, *22*, 523–536, doi:10.2478/aoas-2021-0058.
37. Plata-Pérez, G.; Angeles-Hernandez, J.C.; Morales-Almaráz, E.; Del Razo-Rodríguez, O.E.; López-González, F.; Peláez-Acero, A.; Campos-Montiel, R.G.; Vargas-Bello-pérez, E.; Vieyra-Alberto, R. Oilseed Supplementation Improves Milk Composition and Fatty Acid Profile of Cow Milk: A Meta-Analysis and Meta-Regression. *Animals* **2022**, *12*, doi:10.3390/ani12131642.
38. Singh, A.; Nayak, S.; Baghel, R.; Khare, A.; Malapure, C.D.; Thakur, D.; Sharma, P.; Singh, B.P. Dietary manipulations to alter milk fat composition. *J. Entomol. Zool. Stud.* **2018**, *6*, 176–181.
39. Qureshi, M.S.; Azeemi, T.A. Dietary Manipulations for Enhancing Cardio-Protective Fatty Acids in the Milk of Dairy Cows. **2012**, 423–429.
40. Saran Netto, A.; Silva, T.H.; Martins, M.M.; Vidal, A.M.C.; Salles, M.S.V.; Roma Júnior, L.C.; Zanetti, M.A. Inclusion of Sunflower Oil, Organic Selenium, and Vitamin E on Milk Production and Composition, and Blood Parameters of Lactating Cows. *Animals* **2022**, *12*, doi:10.3390/ani12151968.
41. Baumgard, L.H.; Sangster, J.K.; Bauman, D.E. Milk fat synthesis in dairy cows is progressively reduced by increasing supplemental amounts of trans-10, cis-12 conjugated linoleic acid (CLA). *J. Nutr.* **2001**, *131*, 1764–1769, doi:10.1093/jn/131.6.1764.
42. Sterk, A.; Van Vuuren, A.M.; Hendriks, W.H.; Dijkstra, J. Effects of different fat sources, technological forms and characteristics of the basal diet on milk fatty acid profile in lactating dairy cows-a meta-analysis. *J. Agric. Sci.* **2012**, *150*, 495–517, doi:10.1017/S0021859611000979.
43. Sterk, A. *Ruminal fatty acid metabolism*; 2011; ISBN 9789461730206.
44. Mollica, M.P.; Trinchese, G.; Cimmino, F.; Penna, E.; Cavaliere, G.; Tudisco, R.; Musco, N.; Manca, C.; Catapano, A.; Monda, M.; et al. Milk fatty acid profiles in different animal species: Focus on the potential effect of selected pufas on metabolism and brain functions. *Nutrients* **2021**, *13*, doi:10.3390/nu13041111.
45. Bainbridge, M.L. Enhancing the content of bioactive fatty acids in bovine milk for human health promotion and disease prevention. **2017**, 1–309.
46. Gross, J.; Van Dorland, H.A.; Bruckmaier, R.M.; Schwarz, F.J. Milk fatty acid profile related to energy balance in dairy cows. *J. Dairy Res.* **2011**, *78*, 479–488, doi:10.1017/S0022029911000550.
47. Toral, P.G.; Monahan, F.J.; Hervas, G.; Frutos, P.; Moloney, A.P. Review: Modulating ruminal lipid metabolism to improve the fatty acid composition of meat and milk. challenges and opportunities. *Animal* **2018**, *12*, S272–S281, doi:10.1017/S1751731118001994.
48. Palmquist, D.L.; Lock, A.L.; Shingfield, K.J.; Bauman, D.E. Biosynthesis of Conjugated Linoleic Acid in Ruminants and Humans. *Adv. Food Nutr. Res.* **2005**, *50*, 179–217, doi:10.1016/S1043-4526(05)50006-8.
49. Kokić, B.; Rakita, S.; Vujetić, J. Impact of Using Oilseed Industry Byproducts Rich in Linoleic and Alpha-Linolenic Acid in Ruminant Nutrition on Milk Production and Milk Fatty Acid Profile. *Animals* **2024**, *14*, 1–19, doi:10.3390/ani14040539.
50. Sylvie; Sedláková, K.; Krížová, L.; Hadrová, S.M. Alternative and Unconventional Feeds in Dairy

Diets and Their Effect on Fatty Acid Profile and Health Properties of Milk Fat. *Animlas* **2021**, doi:10.3390/ANI11061817.

51. Zhang, L.; García-Cano, I.; Jiménez-Flores, R. Effect of milk phospholipids on the growth and cryotolerance of lactic acid bacteria cultured and stored in acid whey-based media. *JDS Commun.* **2020**, *1*, 36–40, doi:10.3168/jdsc.2020-0007.
52. Mele, M. Designing milk fat to improve healthfulness and functional properties of dairy products: From feeding strategies to a genetic approach. *Ital. J. Anim. Sci.* **2009**, *8*, 365–373, doi:10.4081/ijas.2009.s2.365.
53. Conte, G.; Serra, A.; Mele, M. *Dairy Cow Breeding and Feeding on the Milk Fatty Acid Pattern*; Elsevier Inc., 2017; ISBN 9780128097625.
54. Kliem, K.E.; Humphries, D.J.; Reynolds, C.K.; Morgan, R.; Givens, D.I. Effect of oilseed type on milk fatty acid composition of individual cows, and also bulk tank milk fatty acid composition from commercial farms. *Animal* **2017**, *11*, 354–364, doi:10.1017/S1751731116001403.
55. Gebereyowhans, S. Potential strategies to enhance conjugated linoleic acid content of milk and dairy products: A review. *Heliyon* **2024**, *10*, e38844, doi:10.1016/j.heliyon.2024.e38844.
56. Mohan, M.S.; O'Callaghan, T.F.; Kelly, P.; Hogan, S.A. Milk fat: opportunities, challenges and innovation. *Crit. Rev. Food Sci. Nutr.* **2020**, *0*, 1–33, doi:10.1080/10408398.2020.1778631.
57. Ghasemi, E.; Golabadi, D.; Piadeh, A. Effect of supplementing palmitic acid and altering the dietary ratio of n-6:n-3 fatty acids in low-fibre diets on production responses of dairy cows. *Br. J. Nutr.* **2021**, *126*, 355–365, doi:10.1017/S0007114520004183.
58. Nudda, A.; Battacone, G.; Neto, O.B.; Cannas, A.; Helena, A.; Francesconi, D.; Atzori, A.S.; Pulina, G. Revista Brasileira de Zootecnia Invited Review Feeding strategies to design the fatty acid profile of sheep milk and cheese. **2014**, *43*, 445–456. https://doi.org/10.1590/S1516-35982014000800008
59. Givens, D.I.; Kliem, K.E. *Improving the nutritional quality of milk*; Woodhead Publishing Limited, 2009; ISBN 9781845693428.
60. Machado, H.V.N.; Pereira, J.C.; Bettero, V.P.; Leonel, F. de P.; Araújo, R.P.; Moreira, L.M.; Teixeira, R.B.; Zervoudakis, J.T. Influence of lipid supplementation on milk components and fatty acid profile. *Rev. Bras. Zootec.* **2017**, *46*, 910–916, doi:10.1590/S1806-92902017001200006.
61. Morales-Almaráz, E.; Soldado, A.; González, A.; Martínez-Fernández, A.; Domínguez-Vara, I.; De La Roza-Delgado, B.; Vicente, F. Improving the fatty acid profile of dairy cow milk by combining grazing with feeding of total mixed ration. *J. Dairy Res.* **2010**, *77*, 225–230, doi:10.1017/S002202991000004X.
62. Chilliard, Y.; Glasser, F.; Ferlay, A.; Bernard, L.; Rouel, J.; Martin, B.; Martin, C.; Enjalbert, F.; Schmidely, P. Que peut-on attendre des pratiques d'élevage pour améliorer la qualité nutritionnelle des matières grasses du lait bovin et caprin ? *OCL - Ol. Corps Gras Lipides* **2010**, *17*, 22–29, doi:10.1684/ocl.2010.0284.
63. Mk, T. Effect of Nutrition on Production, Composition, Fatty acids and Nutraceutical Properties of Milk. *Adv. Dairy Res.* **2015**, *02*, doi:10.4172/2329-888x.1000115.
64. Sueli Freitas dos Santos; Fonteles, N.L. de O.; Sousa, R.T. de; Gonçalves, J. de L.; Barbosa, J. dos S.R.; Santos, S.F. dos; Bomfim, M.A.D. Inclusão de gordura na alimentação de caprinos e seu feito sobre o perfil lipídico no leite: Revisão. *Pubvet* **2016**, *10*, 343–351, doi:10.22256/pubvet.v10n4.343-351.
65. Hanus, O.; Samkova, E.; Křížova, L.; Hasoňova, L.; Kala, R. Role of fatty acids in milk fat and the influence of selected factors on their variability—a review. *Molecules* **2018**, *23*, 1–32, doi:10.3390/molecules23071636.
66. Hillbrick, G. and A.M.A. Deakin Research Online Online. *2007, Interrelat. between Innov. Mark. Orientat. SMEs, Manag. Res. news, vol. 30, no. 12, pp. 878-891.* **2007**, *30*, 878–891.
67. Kennelly, J.J. The fatty acid composition of milk fat as influenced by feeding oilseeds. *Anim. Feed Sci. Technol.* **1996**, *60*, 137–152, doi:10.1016/0377-8401(96)00973-X.

68. Barbulova, A.; Colucci, G.; Apone, F. New trends in cosmetics: By-products of plant origin and their potential use as cosmetic active ingredients. *Cosmetics* **2015**, *2*, 82–92, doi:10.3390/cosmetics2020082.
69. Moran, C.A.; Morlacchini, M.; Keegan, J.D.; Warren, H.; Fusconi, G. Dietary supplementation of dairy cows with a docosahexaenoic acid-rich thraustochytrid, Aurantiochytrium limacinum: Effects on milk quality, fatty acid composition and cheese making properties. *J. Anim. Feed Sci.* **2019**, *28*, 3–14, doi:10.22358/jafs/105105/2019.
70. Markey, O.; Kliem, K.E. *Does modifying dairy fat composition by changing the diet of the dairy cow provide health benefits?*; Elsevier Inc., 2020; ISBN 9780128156032.
71. Lock, A.L.; Garnsworthy, P.C. Independent effects of dietary linoleic and linolenic fatty acids on the conjugated linoleic acid content of cows' milk. *Anim. Sci.* **2002**, *74*, 163–176, doi:10.1017/s1357729800052334.
72. Doreau, M.; Bauchart, D.; Chilliard, Y. Enhancing fatty acid composition of milk and meat through. *Differences* **2011**, *2009*, 19–29.
73. Nudda, A.; Battacone, G.; Neto, O.B.; Cannas, A.; Francesconi, A.H.D.; Atzori, A.S.; Pulina, G. Feeding strategies to design the fatty acid profile of sheep milk and cheese. *Rev. Bras. Zootec.* **2014**, *43*, 445–456, doi:10.1590/S1516-35982014000800008.
74. Lock, A.L.; Bauman, D.E. Modifying milk fat composition of dairy cows to enhance fatty acids beneficial to human health. *Lipids* **2004**, *39*, 1197–1206, doi:10.1007/s11745-004-1348-6.
75. COZMA, A.; MIERE, D.; FILIP, L.; BANC, R.; STANCIU, O.; ANDREI, S.; LOGHIN, F. Factors Influencing the Concentration of Certain Liposoluble Components in Cow and Goat Milk: A Review. *Not. Sci. Biol.* **2014**, *6*, 267–272, doi:10.15835/nsb639404.
76. Carrara, E.R.; Gaya, L.G. and Mourão, G.B. Fatty acid profile in bovine milk: Its role in human health and modification by selection. *Arch. Zootec.* **2016**, *65*, 581–584.
77. Samková, E.; Špička, J.; Pešek, M.; Pelikánová, T.; Hanuš, O. Animal factors affecting fatty acid composition of cow milk fat: A review. *S. Afr. J. Anim. Sci.* **2012**, *42*, 83–100, doi:10.4314/sajas.v42i2.1.
78. Contarini, G.; Toppino, P.M.; Leardi, R.; Polidori, F.; Savoini, G.; Bertocchi, L. Lipid Supplementation of Dairy Cows' Diets: Effects on Milk Fat Composition. *J. Agric. Food Chem.* **1996**, *44*, 3507–3511, doi:10.1021/jf960022y.
79. Oliveira, R.; Faria, M.; Silva, R.; Bezerra, L.; Carvalho, G.; Pinheiro, A.; Simionato, J.; Leão, A. Fatty acid profile of milk and cheese from dairy cows supplemented a diet with palm kernel cake. *Molecules* **2015**, *20*, 15434–15448, doi:10.3390/molecules200815434.
80. Dewhurst, R.J. Targets for milk fat research: Nutrient, nuisance or nutraceutical? *J. Agric. Sci.* **2005**, *143*, 359–367, doi:10.1017/S0021859605005514.
81. Chilliard, Y.; Ferlay, A.; Mansbridge, R.M.; Doreau, M. Ruminant milk fat plasticity: Nutritional control of saturated, polyunsaturated, trans and conjugated fatty acids. *Anim. Res.* **2000**, *49*, 181–205, doi:10.1051/animres:2000117.
82. Duncan, S.E.; Keenan, T.W.; Mcgilliard, M.L.; Vinson, W.E.; Webb, K.E. Modifying Fatty Acid Composition of Bovine Milk. **1997**.
83. Lock, A.L.; Givens, D.I.; Bauman, D.E. Dairy fat: Perceptions and realities. *Milk Dairy Prod. as Funct. Foods* **2014**, 174–197, doi:10.1002/9781118635056.ch6.
84. Lee, S.W.; Chouinard, Y.; Van, B.N. Conjugated linoleic acids alter milk fatty acid composition and inhibit milk fat secretion in dairy cows. *Asian-Australasian J. Anim. Sci.* **2006**, *19*, 799–805.
85. German, J.B.; Dillard, C.J. Composition, structure and absorption of milk lipids: A source of energy, fat-soluble nutrients and bioactive molecules. *Crit. Rev. Food Sci. Nutr.* **2006**, *46*, 57–92, doi:10.1080/10408690590957098.
86. Aleksejeva, S.; Ciprovica, I.; Meija, L. a Review: Dairy Phospholipids in Human Nutrition. *Res. Rural Dev.* **2022**, *37*, 89–92, doi:10.22616/rrd.28.2022.013.
87. Clapperton, J.L.; Kelly, M.E.; Banks, J.M.; Rook, J.A.F. The production of milk rich in protein and low

in fat, the fat having a high polyunsaturated fatty acid content. *J. Sci. Food Agric.* **1980**, *31*, 1295–1302, doi:10.1002/jsfa.2740311212.
88. Lacasse, P.; Kennelly, J.J.; Delbecch, L.; Ahnadi, C.E. Addition of protected and unprotected fish oil to diets for dairy cows. I. Effects on the yield, composition and taste of milk. *J. Dairy Res.* **2002**, *69*, 511–520, doi:10.1017/s0022029902005770.
89. Vasilopoulou, D. Impact of partial replacement of saturated with monounsaturated fatty acids in dairy foods on markers of cardiovascular risk Dafni Vasilopoulou A thesis is part fulfilment of the requirement for the degree of Doctor of Philosophy. **2017**.
90. Wright, T.C.; Holub, B.J.; McBride, B.W. Apparent transfer efficiency of docosahexaenoic acid from diet to milk in dairy cows. *Can. J. Anim. Sci.* **1999**, *79*, 565–568, doi:10.4141/A98-088.
91. Shorten, P.R.; Pleasants, T.B.; Upreti, G.C. A mathematical model for mammary fatty acid synthesis and triglyceride assembly: The role of stearoyl CoA desaturase (SCD). *J. Dairy Res.* **2004**, *71*, 385–397, doi:10.1017/S0022029904000354.
92. Neville, M.C.; Picciano, M.F. Regulation of milk lipid secretion and composition. *Annu. Rev. Nutr.* **1997**, *17*, 159–184, doi:10.1146/annurev.nutr.17.1.159.
93. J. E. STORRY, P. E. BRUMBY*, B. TUCKLEY, V. A. WELCH, D.S.; FULFORD, A.R.J. Effect of feeding protected lipid to dairy cows in early lactation on the composition of blood lipoproteins and secretion of fatty acids in milk. *J. Agric. Sci.* **1980**, *94*, 503–516, doi:10.1017/S0021859600028495.
94. Chaudhary, R.; Rai, S.; Sailo, L.; Farooq, U. Bin; Singh, A.; Naha, B.C.; Kumar, A. Genetic and Non-Genetic Factors Influencing Fatty Acid Composition of Dairy Milk: A Review. *Indian J. Anim. Nutr.* **2017**, *34*, 1, doi:10.5958/2231-6744.2017.00001.9.
95. Roca Fernandez, A.I.; Gonzalez Rodriguez, A. Effect of Dietary and Animal Factors on Milk Fatty Acids Composition of Grazing Dairy Cows: A Review. *Iran. J. Appl. Anim. Sci.* **2012**, *2*, 97–109.
96. Movaliya, J.K.; Dutta, K.S.; Savsani, H.H.; Patil, S.S. Nutritional strategy for designer milk with fat constituents beneficial for human health – A review. *Agric. Rev.* **2014**, *35*, 42, doi:10.5958/j.0976-0741.35.1.005.
97. Chouinard, P.Y.; Corneau, L.; Barbano, D.M.; Metzger, L.E.; Bauman, D.E. Conjugated linoleic acids alter milk fatty acid composition and inhibit milk fat secretion in dairy cows. *J. Nutr.* **1999**, *129*, 1579–1584, doi:10.1093/jn/129.8.1579.
98. C Rymer, K W J Wahle, D.I.G. Dietary strategies for increasing docosahexaenoic acid (DHA) and eicosapentaenoic acid (EPA) concentrations in bovine milk: a review. *CABI Rev.* **2003**, doi:10.1079/PAVSNNR20033059692.
99. Nudda, A.; Cannas, A.; Correddu, F.; Atzori, A.S.; Lunesu, M.F.; Battacone, G.; Pulina, G. Sheep and goats respond differently to feeding strategies directed to improve the fatty acid profile of milk fat. *Animals* **2020**, *10*, 1–22, doi:10.3390/ani10081290.
100. Markey, O.; Vasilopoulou, D.; Givens, D.I.; Lovegrove, J.A. Dairy and cardiovascular health: Friend or foe? *Nutr. Bull.* **2014**, *39*, 161–171, doi:10.1111/nbu.12086.
101. Muñoz-Alvarez KY, Gutiérrez-Aguilar R, F.M. Metabolic effects of milk fatty acids. *Nutr Bull.* **2024**, *49*, 19–39, doi:doi: 10.1111/nbu.12657.
102. Kliem, K.E.; Shingfield, K.J. Manipulation of milk fatty acid composition in lactating cows: Opportunities and challenges. *Eur. J. Lipid Sci. Technol.* **2016**, *118*, 1661–1683, doi:10.1002/ejlt.201400543.
103. Lei, J.; He, Y.; Zhu, S.; Shi, J.; Tan, C.; Liu, Y.; Xu, Y. approach for polyunsaturated fatty acids in milk †. **2024**, 751–760, doi:10.1039/d3an01536j.
104. Aresta, A.; Santis, S. De; Carocci, A.; Barbarossa, A.; Ragusa, A.; Vietro, N. De; Clodoveo, M.L.; Corbo, F.; Zambonin, C. Determination of Commercial Animal and Vegetable Milks ' Lipid Profile and Its Correlation with Cell Viability and Antioxidant Activity on Human Intestinal Caco-2 Cells. **2021**.

105. Ali, A.H.; Khalifa, Salah A., Ren-You Gan, Nagendra Shah, M.A. Fatty acids, lipid quality parameters, and amino acid profiles of unripened and ripened cheeses produced from different milk sources. *J. Food Compos. Anal.* **2023**, *123*, doi:https://doi.org/10.1016/j.jfca.2023.105588.
